# A cross-tissue physicochemical causal chain underlying vertebrate mandibular morphogenesis

**DOI:** 10.1126/sciadv.aec7997

**Published:** 2026-05-08

**Authors:** Kazutaka Hosoda, Daisuke Ohtsuka, Sang-Woo Lee, Naofumi Kawahira, Toru Kawanishi, Yoshihiro Morishita

**Affiliations:** ^1^ Laboratory for Developmental Morphogeometry, RIKEN Center for Biosystems Dynamics Research, Kobe 650-0047, Japan.; ^2^ Department of Molecular, Cell, and Developmental Biology, School of Life Sciences, University of California, Los Angeles, Box 951606, 490D BSRB, Los Angeles, CA 90095, USA.; ^3^ School of Science and Technology, Institute of Science Tokyo, Kanagawa, 226-8501, Japan.

## Abstract

Understanding morphogenesis increasingly requires attention to physical aspects. In complex embryonic structures comprising multiple adjacent tissues, morphogenetic change in one tissue can impose geometric constraints that shape another tissue. However, such cross-tissue physical coordination remains largely unexplored. Using zebrafish jaw development, we show that dynamic folding of the oral ectoderm drives the elongation and fusion of mandibular primordia by constraining and guiding mesenchymal cell motion. This folding is initiated by Shh signaling, mainly from the adjacent neural tube, which establishes polarized behaviors in oral ectodermal cells. Notably, this phase of mandibular formation proceeds independently of cell proliferation and relies on polarized motion of both epithelium and mesenchyme. Our findings identify a physicochemical causal chain, offering a holistic view of complex embryonic tissue shaping.

## Teaser

Folding of oral ectoderm acts as a driver that coordinates mesenchymal dynamics in vertebrate jaw formation.

## INTRODUCTION

A central challenge in developmental biology is to understand how complex organs reproducibly acquire their three-dimensional (3D) shapes during embryogenesis. Although genetic programs and molecular signaling pathways provide essential instructions for development (*1*, *2*), growing evidence indicates that morphogenesis cannot be fully explained by biochemical regulation alone. In particular, the physical context in which cells and tissues move, deform, and interact plays a critical role in shaping developing organs (*3*–*14*). Recent advances in live imaging and quantitative analysis have begun to illuminate how collective cell motion, tissue mechanics, and geometric factors contribute to self-organizing morphogenetic processes, particularly in relatively simple or isolated tissue contexts.

In parallel with biochemical regulation, several recent studies have highlighted the importance of physical interactions between adjacent tissues in driving tissue shape changes. These include mechanical instabilities arising from differential growth between physically coupled tissues (*15*–*17*), frictional interactions that transmit forces across tissue interfaces (*18*), and confinement imposed by relatively static external boundaries (*19*, *20*). Such mechanisms have provided valuable insights into how physical forces and constraints influence morphogenesis. In most of these systems, the geometry of tissue-tissue contact or surrounding boundaries is largely fixed or externally imposed, allowing physical interactions to be analyzed under relatively stable geometric conditions.

In contrast, developing embryos exhibit a much higher level of complexity. Multiple tissues are three-dimensionally intertwined and undergo large-scale deformations in a highly coordinated manner. As morphogenesis proceeds, the geometry of tissue-tissue interfaces and the spatial constraints experienced by cells are continuously reconfigured by the tissues themselves. How dynamically evolving spatial constraints are generated by underlying molecular and cellular dynamics and how the time-evolving morphological changes of one tissue act as spatial constraints for adjacent tissues to influence their morphogenesis remain poorly understood, in part because such processes are difficult to isolate and manipulate experimentally. Addressing this gap is essential for understanding complex 3D morphogenesis in multitissue systems.

Here, we investigate this issue using mandibular morphogenesis in zebrafish embryos as a model system. During early craniofacial development, the left and right mandibular mesenchymal primordia undergo robust elongation and midline fusion while remaining closely associated with an adjacent epithelial tissues, the ventral oral ectoderm (vOE), providing a tractable system to study cross-tissue interactions in three dimensions. By combining single-cell–resolution 4D imaging, quantitative analysis of collective cell motion and tissue deformation, and targeted genetic and chemical perturbations, we delineate a causal sequence in which Shh-dependent cell polarity in the vOE drives cell division–independent polarized collective motion and epithelial folding, which is required for subsequent elongation and fusion of the mandibular mesenchymal primordia.

Our analyses further show that epithelial folding reshapes the spatial environment surrounding the mesenchyme and is associated with biased mesenchymal expansion, consistent with a role for dynamically evolving spatial constraints in guiding mesenchymal morphogenesis. Given that similar epithelial-mesenchymal architectures are observed in multiple organs, our findings may provide broader insights into general principles governing multitissue morphogenesis. By highlighting how dynamically evolving tissue geometry can influence morphogenesis across tissue boundaries, this work contributes to a more integrated understanding of embryonic development beyond individual organ systems.

## RESULTS

### Region-specific tissue deformation of PA1

**Fig. 1.**
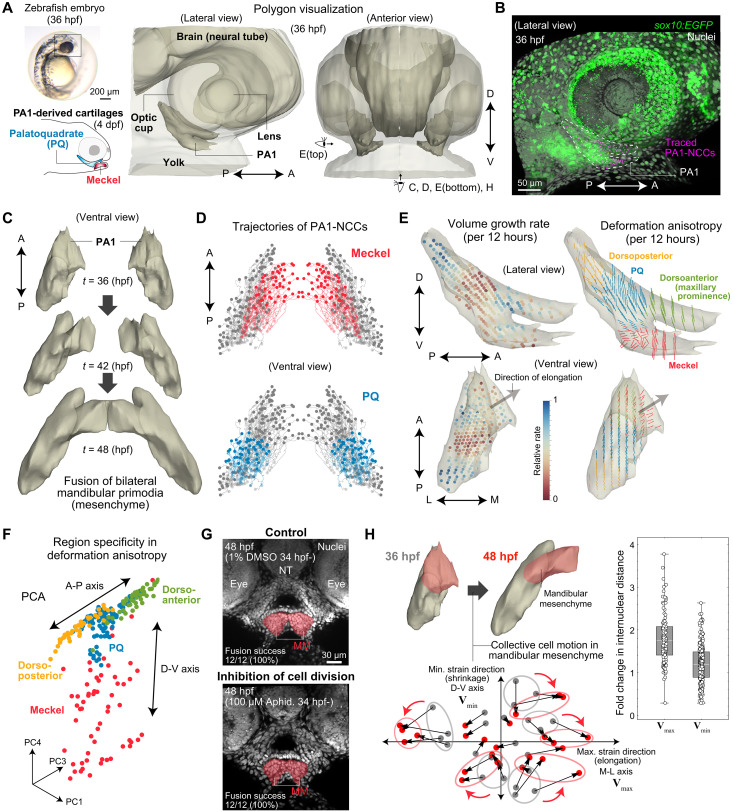
Live imaging and data analysis reveal region-dependent tissue and cell dynamics within PA1. (**A**) (Top left) A zebrafish embryo. (Bottom left) Schematic of PA1-derived jaw cartilages at 4 days postfertilization (dpf). (Right) Polygonal representation of the head at 36 hpf. Viewing directions are indicated: ventral for (C), (D), (E, bottom), and (H); lateral for (E, top). (**B**) Time-lapse imaging of jaw development. Magenta spots indicate traced PA1-NCCs. (**C**) Ventral views showing morphological changes in PA1 during elongation and fusion of bilateral mandibular mesenchymal primordia. (**D**) PA1-NCC lineage trajectories (36 to 48 hpf), mirrored across the midline. Presumptive Meckel’s (red) and PQ (blue) cartilage cells were retrospectively identified from final positions. (**E**) Spatial maps of volume growth rate (left) and deformation anisotropy (right) between 36 and 48 hpf, shown within the 36 hpf PA1 polygon. Colored dots indicate relative volume change over 12 hours; segments denote the maximum deformation direction. (**F**) Principal components analysis (PCA) of the deformation tensor eigenvalues and eigenvectors reveals regional differences in anisotropy between dorsal and ventral PA1. (**G**) Transverse sections show normal fusion of bilateral mandibular mesenchyme (MM, red) in both control [dimethyl sulfoxide (DMSO)] and aphidicolin-treated embryos, despite inhibited proliferation in the latter. NT, neural tube. (**H**) Quantification of collective motion of presumptive Meckel’s cells. (Bottom left) Representative relative positional changes from 36 hpf (gray) to 48 hpf (red) within a single embryo, illustrating a clear pattern of polarized rearrangement consistent with the quantified M-L tissue elongation shown in the graphs (right). (Right) Fold changes in internuclear distances for cell pairs aligned along the maximum (**V**_max_) and minimum (**V**_min_) deformation axes. Polygons were mirrored across the midplane in (A) and (C). See Materials and Methods for details on mathematical/statistical analyses. A-P, antero-posterior; D-V, dorsoventral.

The jaw is the earliest-forming skeletal structure among craniofacial elements and provides a well-defined yet sufficiently complex system for studying tissue dynamics and cross-tissue interactions. While transcription factors responsible for regionalization and various morphogens are known to influence jaw primordium size and morphology (*8*, *21*–*35*), the physical processes underlying tissue deformation, collective cell movement, and their driving forces remain poorly understood. To investigate tissue and cellular dynamics during jaw morphogenesis, we performed 4D live imaging of the first pharyngeal arch (PA1) in transgenic zebrafish expressing fluorescent proteins under the *sox10* promoter, labeling neural crest–derived cells (NCCs), which constitute much of the PA1 mesenchyme (Fig. 1, A and B, and movie S1). We focused on the period from 36 to approximately 48 hours postfertilization (hpf), which spanned the interval from the establishment of gene expression patterns (*36*) to midline fusion of the ventral portions of the bilateral PA1s, forming the mandible (Fig. 1C). Although the broad fate of PA1-NCCs was known, their detailed regional origin remained unclear. By tracing cell trajectories back from 48 hpf, when morphological regionalization and cell aggregation before cartilage differentiation become evident, we identified the origins of future Meckel’s and palatoquadrate (PQ) cartilages and other dorsal NCC populations (Fig. 1D). Notably, the Meckel’s cartilage region corresponded to *hand2* and *dlx5/6* expression, while the PQ region aligned with dorsal *notch* expression, as previously reported (*32*, *37*–*40*).

To analyze global morphogenetic dynamics, we reconstructed 3D tissue deformation maps of PA1 mesenchyme from cell trajectory data using a Gaussian process model, a Bayesian regression framework. This approach enables smooth and continuous deformation estimation while accounting for stochastic noise from cell movement, tracking limitations, and inter-embryo variability (fig. S1A and Materials and Methods). From this, we calculated the local volume growth rate and deformation anisotropy across PA1 (Fig. 1E and fig. S1B). The volume growth rate was significantly lower in the presumptive Meckel’s and PQ cartilage regions compared to surrounding areas (Fig. 1E, left, and fig. S1C). However, no clear differential growth along the axis of elongation was observed within the mandibular primordia across embryos. In contrast, deformation anisotropy showed clear region-specific patterns (Fig. 1E, right). While dorsoventral (D-V) thickening occurred broadly, elongation along other axes varied: Anterior-posterior (A-P) elongation was predominant in the dorsal PA1, whereas mediolateral (M-L) elongation toward the facial midline was characteristic of the medioventral region, corresponding to a part of presumptive Meckel’s cartilage (movie S1). These patterns were supported by principal components analysis of local deformation anisotropy, which also highlighted a D-V distinction within PA1 (Fig. 1F), and were reproducible across multiple embryos (fig. S1, D and E).

In summary, 4D imaging and quantitative analysis revealed region-specific deformation dynamics within PA1 mesenchyme. Notably, anisotropic tissue elongation along the M-L axis in the presumptive Meckel’s cartilage region provides a physical basis for the midline fusion of the bilateral mandibular primordia.

### Mandibular mesenchyme morphogenesis by polarized cell motion, not cell division

Next, we examined the cellular mechanisms underlying anisotropic tissue deformation in ventral PA1, a process essential for mandibular formation. Although previous clonal analysis had suggested a role for oriented cell division in mandibular formation (*41*), our quantification revealed that division orientation was biased nearly perpendicular to the elongation axis (fig. S1F), ruling it out as a driving factor. In addition, inhibiting cell proliferation with aphidicolin did not impair mandibular primordia fusion—100% of embryos showed successful fusion (Fig. 1G and fig. S2)—although later-stage inhibition reduced Meckel’s cartilage (fig. S1G), consistent with the previous report (*41*). These observations indicate that cell proliferation, including its orientation, is not required for the elongation and midline fusion of the mandibular primordia during early mandible morphogenesis. We also assessed cell shape by segmenting cell boundaries. The long axes of these cells tended to align perpendicularly to the tissue elongation direction, consistent with the division orientation, and this pattern remained stable over time (fig. S1H). Thus, cell shape changes also do not contribute to the observed anisotropic deformation.

Next, we examined whether anisotropic deformation in the mandibular mesenchyme (medioventral PA1) arises from cell rearrangements. By projecting cell positions onto the plane defined by the axes of maximal stretch and shrinkage, we observed a clear pattern: Cells converged along the shrinkage axis and diverged along the stretch axis (Fig. 1H and Materials and Methods). To quantify this, we measured 12-hour changes in nuclear distances for neighboring cell pairs that were aligned along the ML or DV axis at both 36 and 48 hpf. ML-aligned pairs showed significant separation along the ML direction (1.7-fold), whereas DV-aligned pairs remained largely unchanged (1.1-fold). These results indicate that anisotropic deformation of the mandibular mesenchyme is primarily driven by cell rearrangements. Together with our findings on cell division orientation and cell shape, we conclude that the elongation of the mandibular primordia (up to bilateral fusion) is primarily driven by polarized cell rearrangements toward the midline, rather than by cell proliferation or changes in cell shape.

### Dynamic folding of oral ectoderm over the mandibular mesenchyme

**Fig. 2.**
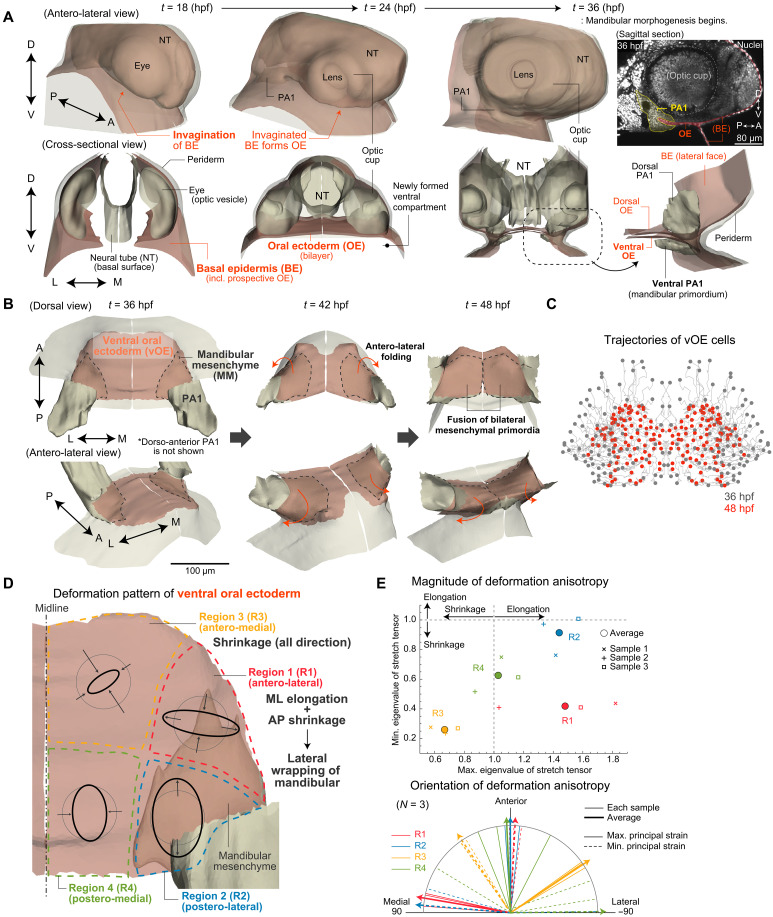
Folding dynamics of the vOE over the mandibular mesenchyme. (**A**) Temporal changes in head region organ and tissue morphologies, represented as polygons reconstructed from microscopy images, at 18, 24, and 36 hpf. BE and OE are shown as red open surfaces. The OE forms by invagination of the BE and comprises dorsal and ventral layers. The PA1 is absent at 18 hpf but becomes evident by 24 hpf; its ventral portion (presumptive Meckel’s region) lies within the ventral compartment formed by the OE. An optical sagittal section at 36 hpf is shown on the right. Nuclei were labeled by tdTomato–NLS mRNA injection. The OE (red) is sandwiched between dorsal and ventral PA1 mesenchyme (yellow). (**B**) Temporal changes in the vOE (red) and mandibular mesenchyme morphologies, visualized as polygons from microscopy images, at 36, 42, and 48 hpf. (**C**) Dorsal view of vOE cell trajectories from 36 to 48 hpf, mirrored across the midline. (**D**) Quantification of local tissue deformation within the vOE (dorsal view). The right half was divided into four subregions. Although vOE cells move in three dimensions, each subregion remains approximately planar during the analyzed time window. Deformations were therefore approximated as 2D linear transformations embedded in 3D space and mapped back onto the predeformation configuration at 36 hpf. The results are visualized as deviations from a circle (inward: shrinkage; outward: elongation). (**E**) Magnitude (top) and orientation (bottom) of deformation anisotropy within the vOE (*N* = 3 embryos). Eigenvalues of the deformation tensor in the posterior half were closer to 1, indicating weaker in-plane deformation than in the anterior half. Polygons were mirrored across the midplane in (A) and (B).

To explore the mechanism behind anisotropic elongation of the mandibular primordia via polarized collective cell motion, we focused on the morphological changes of the adjacent oral ectoderm (OE). Notably, as illustrated in Fig. 2 (A and B), the OE (colored red and forming a curved epithelial sheet) undergoes dynamic and pronounced folding, wrapping the bilateral mandibular mesenchyme during their elongation and fusion at the midline (see also movie S2). This observation led us to hypothesize a potential causal link between OE folding and mandibular morphogenesis.

OE has been suggested to contribute to PA1 positioning by attracting cranial NCCs through platelet-derived growth factor signaling (*42*) or stromal cell–derived factor 1 (SDF-1)/CXCR4 signaling (*43*). It is thought to originate from the invagination of the cranial basal epidermis (BE) (*44*–*47*), which we confirmed via live imaging and polygonal reconstruction as follows (Fig. 2A). Around 14 hpf, when neurulation is completed, the BE begins invaginating near the yolk neural tube (NT) boundary (fig. S3), resulting in the formation of a bilayered epithelium (i.e., oral ectoderm) by 24 hpf. This process establishes a dorsal-ventral partition of the face that defines a new ventral compartment where the mandible will form (Fig. 2A). At 36 hpf, when mandibular primordia start elongating, the OE lies flat and is sandwiched by PA1 on its anterior side (Fig. 2A). The OE consists of two cell layers, dorsal and ventral, which undergo distinct morphological changes. Notably, the ventral OE (vOE) folds to wrap the bilateral mandibular primordia by 48 hpf, when they fuse (Fig. 2B and movie S2).

To investigate the causal relationship between vOE folding and mandibular morphogenesis, we began by quantifying vOE tissue and cell dynamics as a basis for analyzing its response to perturbations. Given the small number of cells constituting the vOE, we did not reconstruct its deformation map as a continuum. Instead, we divided vOE into four regions by bisecting it along the M-L and A-P axes and approximated tissue deformation in each region by a linear transformation fitted to collective cell displacements. This analysis revealed clear regional differences, consistent across embryos (*N* = 3; Fig. 2, C to E). In particular, the anterior half exhibited greater deformation. In the antero-lateral region, where peripheral folding occurs toward antero-lateral direction to wrap the mandibular mesenchyme, strong deformation anisotropy was evident, characterized by A-P shrinkage and M-L elongation. The antero-medial region undergoes shrinkage in all directions, which may cause the folding points on both sides to shift further medially (Fig. 2B). In contrast, the posterior half exhibited modest deformation (Fig. 2E).

**Fig. 3.**
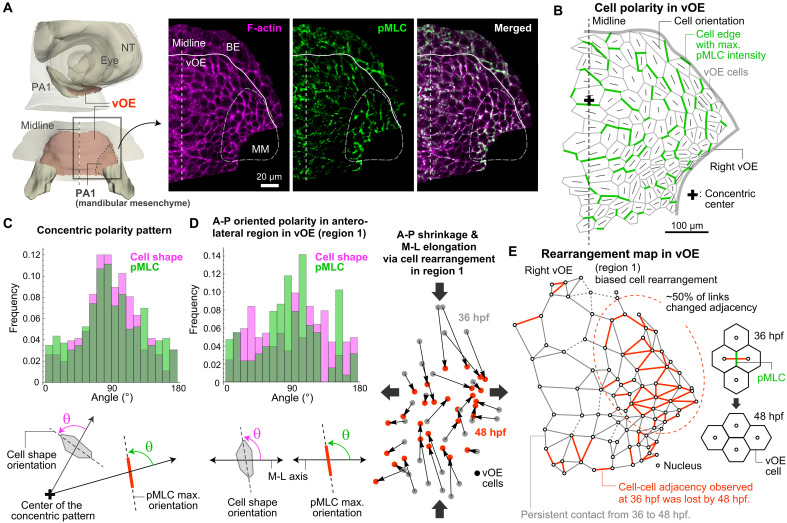
Polarity in actomyosin localization and cell dynamics in the vOE. (**A**) Detection of F-actin and phosphorylated myosin light chain (pMLC) in the vOE at 36 hpf, viewed dorsally. The boundary between the vOE and BE is indicated by solid lines; the mandibular mesenchyme (MM) beneath the vOE is outlined by dashed lines. (**B**) Polarity patterns of cell shape and pMLC localization within the vOE. The thick gray line outlines the right vOE region. The black cross on the midline marks the estimated center of the concentric polarity pattern in cell shape orientation. (**C**) Histograms of cell shape orientation and pMLC localization angle in the vOE. To reveal their concentric patterns, measurements were made relative to the radial axis defined with respect to the estimated center of the concentric polarity pattern (see Materials and Methods for details on center determination) (*N* = 5). (**D**) (Left) Histograms of cell shape orientation (*N* = 6) and pMLC localization angle (*N* = 5) in the anterolateral region (region 1). Measurements were made relative to the M-L axis, revealing A-P–biased polarity. (Right) Analysis of collective cell movement based on nuclear positions of vOE cells in region 1. The relative change in vOE cell positions from 36 hpf (gray) to 48 hpf (red) indicates cellular rearrangements, which drive M-L tissue elongation and A-P shortening. (**E**) Rearrangement map within the vOE. Cell nuclei at 36 hpf are shown as white circles. Links indicate cell-cell adjacency at 36 hpf. Red links represent pairs of cells that lost adjacency by 48 hpf (i.e., due to cell rearrangement). Solid black links represent persistent adjacency, and dashed links indicate adjacency not evaluable at 48 hpf. Anterior is oriented toward the top.

We then investigated the cellular behaviors underlying vOE folding. Notably, vOE folding occurred normally, as did fusion of the bilateral mandibular primordia, even when cell proliferation was inhibited, indicating that the anisotropic deformation of the antero-lateral vOE is proliferation independent (figs. S2C and S4A). Instead, vOE cells exhibit an intriguing polarity pattern: Their longitudinal edges align circumferentially, forming a concentric pattern, and active myosin—a key regulator of junctional contraction and cell rearrangement (*48*, *49*)—localizes along these edges in a similarly oriented fashion (Fig. 3, A to C). In particular, in the anterolateral region (region 1 in Fig. 2D), which exhibits dynamic folding, active myosin localized along the A-P axis, and cells intercalated along this axis, resulting in shrinkage in the A-P direction and elongation in the perpendicular (M-L) direction. Since cell shape orientation remained largely stable during mandibular morphogenesis (fig. S4B), we identified polarized cell rearrangement as the main driver of antero-lateral folding (Fig. 3D and movie S3). Supporting this, frequent cell rearrangements—quantified by neighbor changes—were detected only in the anterolateral region (Fig. 3E). By contrast, tissue shrinkage in the anteromedial region (region 3 in Fig. 2D) primarily resulted from reduced in-plane cell area (fig. S4C), with minimal rearrangement (Fig. 3E). Quantification of cell area changes across vOE subregions showed a similar trend to the tissue-level deformation analysis, with region 3 displaying the most pronounced area shrinkage (fig. S4C). In summary, our analysis of vOE tissue and cell dynamics revealed that (i) the vOE forms via invagination of the cranial BE, establishing the facial D-V compartment; (ii) it folds dynamically during mandibular primordia elongation and fusion, wrapping around both mesenchymal primordia; (iii) this antero-lateral folding is primarily driven by polarized cell rearrangements that induce A-P shortening and M-L elongation in the anterolateral region.

### vOE folding drives mandibular morphogenesis

**Fig. 4.**
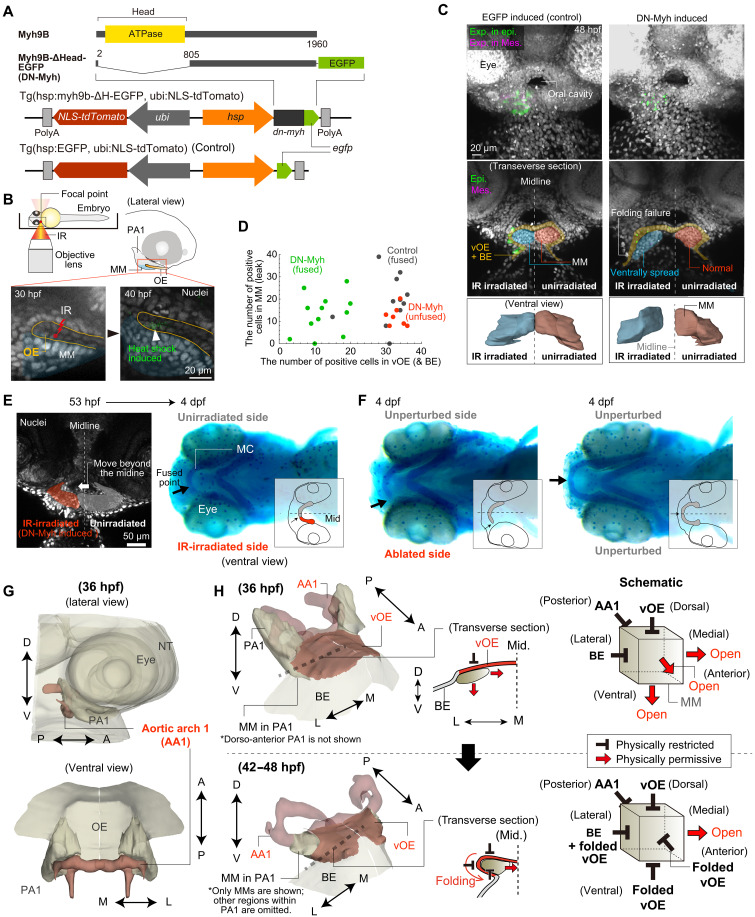
Folding dynamics of the vOE drive mandibular morphogenesis. (**A**) Schematics of the established transgenic (Tg) lines. *Ubi*, ubiquitin B promoter; *hsp*, heat shock promoter. (**B**) Local transgene induction using the infrared–laser evoked gene operator (IR-LEGO) system. Validation in *Tg(hsp:CreERT2-T2A-mCherry; EF1*α*:H2B-EGFP)* embryos showed that heat shock by IR laser irradiation induced mCherry expression (green) in restricted OE cells. (**C**) Unilateral induction of EGFP (control) or DN-Myh by IR laser irradiation. (Top) Maximum intensity projection (anterior view) of images. (Middle) Transverse section. (Bottom) Polygons delineating the mandibular mesenchyme (MM) region (ventral view). DN-Myh expression impaired folding of the vOE and medial movement of the MM on the irradiated side. Green: transgenes expressed in the vOE and surrounding BE; magenta: transgenes expressed in MM (see Materials and Methods for details of fluorescence signal extraction). (**D**) Quantification of cells expressing EGFP (control) or DN-Myh in the vOE/BE (*X* axis) and MM (*Y* axis) following irradiation (DN-Myh: *N* = 19; control: *N* = 11). (**E**) (Left) DN-Myh induction caused left-right asymmetric elongation of the MM at 53 hpf (anterior view). By 4 dpf, the embryo developed left-right asymmetry in Meckel’s cartilage size (ventral view, Alcian Blue; anterior to the left). (**F**) Laser ablation of the right PA1 at 32 hpf and untreated control. (**G**) Polygons delineating head structures at 36 hpf, mirrored across the midline. (**H**) (Right) Schematic of spatial constraints on MM imposed by surrounding tissues and their temporal evolution. (Left) Polygons delineating structures around the MM. (Middle) Transverse sections through the MM at 36 hpf (top) and 42 to 48 hpf (bottom). AA1, aortic arch 1; IR, infrared; MC, Meckel’s cartilage.

Finally, to test the causal role of vOE folding in mandibular morphogenesis, we targeted its actomyosin-dependent cell rearrangement (*11*, *48*, *49*). Treatment of the entire embryo with (-)-blebbistatin, a nonmuscle myosin II inhibitor, disrupted both vOE folding and fusion of the bilateral mandibular primordia (control: 13 of 13 fused; blebbistatin: 0 of 14 fused; fig. S5A; see also fig. S5B, which shows minimal cell rearrangement in the anterolateral vOE under blebbistatin treatment), indicating that myosin activity is essential. For localized inhibition, we generated transgenic zebrafish that express a dominant-negative myosin heavy chain (DN-Myh) upon heat shock [*Tg(hsp:myh9b-*Δ*H-EGFP, ubi:NLS-tdTomato)*] (Fig. 4A, fig. S5C, and Materials and Methods). Heat shock–inducible enhanced green fluorescent protein (EGFP)–only controls were used for comparison (Fig. 4A and fig. S5C).

To induce DN-Myh specifically around the vOE, we used the infrared–laser evoked gene operator (IR-LEGO) system (*50*), which enables localized gene expression via activation of a heat shock promoter (hsp) by spatially focused infrared laser irradiation (Fig. 4B). In this experiment, transgene expression was induced in the right side of the OE, with the untreated left side serving as an internal control. Because the OE is thin and located relatively deep within the embryo, achieving strictly localized induction is technically challenging. Consequently, leaky transgene expression was occasionally observed in adjacent tissues, including the mandibular mesenchyme. Moreover, induction efficiency varied across individuals. We therefore quantified transgene-positive cells in the vOE and surrounding epidermis (*N*_E_), as well as in the mandibular mesenchyme (*N*_M_), for each sample, and examined their association with the resulting phenotype (Fig. 4C).

The results fell into two categories (Fig. 4, C and D). Embryos with fewer DN-Myh–expressing ectodermal cells (*N*_E_ < 30) exhibited normal morphogenesis, including proper vOE folding and mandibular primordia fusion at 48 hpf. In contrast, embryos with more DN-Myh–expressing cells (*N*_E_ > 30) showed impaired folding of the vOE (Fig. 4C), and unfused mandibular primordia at 48 hpf. The number of cells with leaky transgene expression in the mesenchyme (*N*_M_) was not significantly different between the two groups (Fig. 4D). More than 75% of DN-Myh–expressing cells in the vOE were found in region 1 (i.e., anterolateral), while the other regions had only a few cells each (fig. S5D and Materials and Methods). All control embryos with EGFP-only induction exhibited normal development, indicating that the heat shock itself had no effect. These findings demonstrate that mandibular morphogenesis, including the fusion of bilateral primordia, is not mesenchyme autonomous but driven by myosin-dependent folding of the adjacent OE through polarized cell rearrangement.

### vOE folding constrains collective movement of the mandibular mesenchyme

We next asked how vOE folding is related to elongation and fusion of the mandibular mesenchyme. While multiple mechanisms may potentially contribute to mesenchymal morphogenesis, including chemical or extracellular matrix–mediated cues, our imaging analyses reveal a clear spatial relationship between vOE folding and the patterns of mesenchymal cell movement, consistent with the presence of spatial constraints on mesenchymal expansion imposed by surrounding tissues. First, in embryos where folding was inhibited on one side, mandibular mesenchymal cells on that side spread ventrally into the available space instead of extending medially (Fig. 4C). Second, when these embryos were raised to 53 hpf, the mandibular mesenchyme underlying the IR-unirradiated normal vOE extended beyond the midline into the opposite side (Fig. 4E). Furthermore, when one mandibular primordium was severely ablated—an extreme manipulation—the remaining primordium elongated across the midline, nearly reaching the opposite end (Fig. 4F). These findings indicate that mandibular mesenchyme elongation continues as long as space permits, rather than being strictly limited by the midline or following a predetermined trajectory.

Along these lines, the above observations are not readily explained by a model in which mesenchymal elongation is driven primarily by friction or tight physical coupling between the epithelium and mesenchyme. In particular, mandibular mesenchymal cells do not consistently follow epithelial movements but instead expand into available space when such space is present. Moreover, vOE folding occurs predominantly in the lateral direction, whereas the mandibular mesenchyme elongates antero-medially. Consistent with this directional difference, when epithelial and mesenchymal cells at the same M-L level were labeled before mesenchymal elongation, mesenchymal cells were observed to move medially earlier than the overlying epithelial cells (fig. S6). In addition, epithelial cells that were initially in contact with the medial-front mesenchymal cells before elongation were no longer adjacent to these cells at the time of primordia fusion, indicating that medial-front mesenchymal cells advance medially ahead of the overlying epithelium. These observations indicate that epithelial and mesenchymal movements are not tightly coupled during this process. Together, our data are consistent with the idea that vOE folding contributes to the spatial restriction of mesenchymal expansion during mandibular morphogenesis. We note that mesenchymal cell movement is also constrained posteriorly, as the posterior side of PA1 is bordered by relatively large blood vessels, including the aortic arch (Fig. 4G and movie S2).

For clarity, we present a simplified schematic (Fig. 4H) that illustrates how spatial constraints on the mandibular mesenchyme are established by surrounding tissues and evolve over time. At ~36 hpf, the mandibular mesenchyme is bounded dorsally by the vOE, laterally by the facial BE, and posteriorly by large blood vessels (aortic arch), restricting motion in those directions. As vOE folding progresses, the vOE folds antero-laterally and progressively extends to overlie the ventral aspect of the mandibular mesenchyme, thereby establishing additional ventral and anterior boundaries and leaving the medial side as the only permissive direction; this open medial space provides a spatial context consistent with mesenchymal expansion and the subsequent elongation and midline fusion of the bilateral mandibular primordia.

### Neural tube-derived Shh induces vOE polarity and folding

**Fig. 5.**
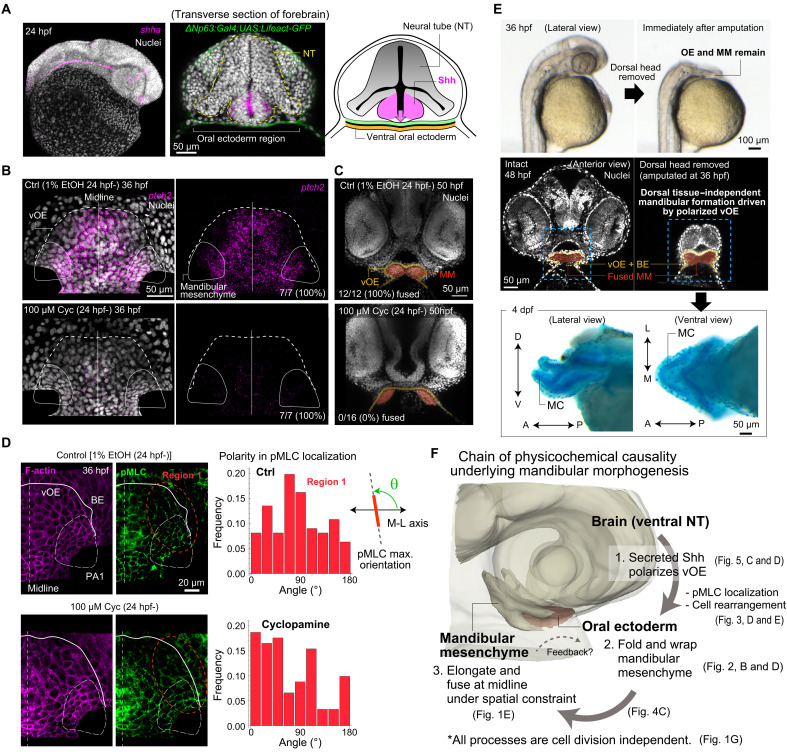
NT-derived Shh induces cell polarity and folding of the vOE. (**A**) *shha* expression at 24 hpf. (**B**) Dorsal view of *ptch2* expression within the vOE (outlined by dashed lines) at 36 hpf. The mandibular mesenchyme located just beneath the vOE is outlined by white closed curves. Shh inhibition by treatment with cyclopamine (Cyc) resulted in near-complete loss of *ptch2* expression. (**C**) Transverse sections of the face in embryos treated with ethanol (control, top) or cyclopamine (bottom) at 50 hpf. (**D**) (Left) Detection of F-actin and pMLC in the vOE of embryos treated with ethanol (control, top) or cyclopamine (bottom). (Right) Polarity of pMLC localization in the anterolateral region (region 1) of the vOE in control (*N* = 6) and cyclopamine-treated (*N* = 5) embryos. Measurements were made relative to the M-L axis. Whereas control embryos show a peak orientation around 90° (i.e., A-P polarity)—nearly perpendicular to the direction of region 1 elongation—cyclopamine-treated embryos exhibit a perturbed orientation pattern. (**E**) (Top) Surgical removal of the dorsal head region in live embryos at 36 hpf. (Middle) Intact and dorsal-head-removed embryos. Nuclei were visualized by injection of tdTomato-NLS mRNA at the one-cell stage. Once cell polarity in the vOE is established, mandibular formation proceeds normally in a self-organized manner, independently of dorsal structures. (Bottom) Alcian blue staining shows that Meckel’s cartilage (MC) forms successfully in embryos that underwent dorsal head removal at 36 hpf. (**F**) The summary diagram illustrating the chain of physicochemical causality underlying mandibular morphogenesis. MM, mandibular mesenchyme; EtOH, ethanol. The A-P, D-V, and M-L axes are indicated by arrows.

Our analysis showed that vOE folding, via polarized cell rearrangement, drives elongation and fusion of the mandibular primordia. This raised the question of what regulates oral ectodermal cell polarity. Here, we focused on Shh signaling from the ventral NT. We did so because (i) in our previous work, we showed that SHH from the ventral NT regulates both the polarity of active-myosin localization and the collective motion of neuroepithelial cells during optic-vesicle formation/elongation in early forebrain development (*11*), and (ii) the NT lies in close proximity to the OE. Consistent with this anatomical rationale, SHH from the ventral NT is known to induce Rathke’s pouch (the pituitary primordium) from the dorsal OE, which directly contacts the ventral NT (*51*). Thus, Shh should readily reach the immediately subjacent vOE as well. To visualize *shh* expression broadly across the head, we performed whole-mount in situ hybridization for *shha* (noting that *shhb* mutants displayed normal phenotypes; 22 of 22 embryos). We found strong *shha* expression in the anteroventral NT, immediately adjacent to the anteromedial vOE where folding occurs (Fig. 5A and fig. S7). *shha* expression was also detectable in the notochord—a known Shh source—but this source lies much farther from the vOE. In the pharyngeal endoderm located posterior to PA1, *shha* expression was detectable but much weaker than in the NT (fig. S7). Last, we confirmed reception of Shh signaling in the vOE by detecting *ptch2* expression, a canonical pathway readout (Fig. 5B).

When embryos were treated with cyclopamine, a Shh signaling inhibitor, *ptch2* expression in the vOE was lost, and both vOE folding and mandibular mesenchyme fusion were inhibited (control: 12 of 12 fused; cyclopamine: 0 of 16; Fig. 5C). Active myosin polarity in the antero-lateral vOE (region 1) was disrupted (Fig. 5D), and cell rearrangements were markedly reduced (fig. S5E). These results demonstrate that Shh-dependent polarity in the vOE is crucial for antero-lateral folding and that loss of this polarity causes folding failure (Fig. 5C). Once Shh-dependent polarity is established in the vOE, the folding of vOE and midline fusion of the bilateral mandibular primordia proceed normally even after surgical removal of dorsal head tissues, including the NT (Fig. 5E). This finding indicates that NT-derived Shh signaling is required not for the continuous maintenance but rather for the initial induction of the self-organizing capacity of the vOE to drive mandibular morphogenesis.

By integrating our analyses, we identified the following physicochemical causal chain underlying mandibular morphogenesis (Fig. 5F): (i) Shh signaling from the ventral forebrain establishes cell polarity in the vOE, including active myosin localization and oriented collective movement. (ii) Frequent polarized rearrangements in the antero-lateral vOE, adjacent to the mandibular mesenchymal primordia, cause M-L elongation and A-P shrinkage of the vOE, inducing antero-lateral folding that wraps around the primordia. (iii) This vOE geometric deformation drives elongation and fusion of the bilateral primordia, forming the mandible. Notably, this entire process occurs independently of cell proliferation.

## DISCUSSION

Morphogenesis of complex organs emerges from coordinated interactions among multiple tissues and is traditionally understood to be governed primarily by chemical signaling pathways, including well-characterized epithelium-mesenchyme interactions (*1*, *52*). In parallel with these biochemical mechanisms, recent works have demonstrated that physical interactions between adjacent tissues can play essential roles in shaping developing organs. In such systems, tissue morphology is influenced by mechanical instabilities arising from differential growth, frictional force transmission between physically coupled tissues, or confinement by relatively static external boundaries, in which the geometry of tissue-tissue contact or surrounding constraints is largely fixed over time.

In contrast, our findings highlight a distinct mode of tissue-tissue interaction during mandibular morphogenesis, in which spatial constraints are not preexisting or externally imposed but are progressively generated through the 3D deformation of interacting tissues themselves. Such dynamically evolving spatial constraints may constitute a critical but previously underappreciated layer of regulation in complex 3D morphogenesis. This mode of regulation is exemplified in mandibular morphogenesis by the dynamic 3D deformations and interaction of both the vOE and the underlying mesenchyme.

While our findings indicate that active folding of the vOE is required for mandibular mesenchymal elongation and midline fusion, it remains difficult to attribute these processes solely to a single mechanism based on dynamically progressing spatial constraints. At present, our observations support a model in which evolving geometric constraints contribute to mesenchymal morphogenesis, but they do not exclude the involvement of additional mechanisms. In particular, the molecular characteristics of the OE derived from the facial BE remain poorly characterized, leaving open the possibility that chemical interactions between tissues—through secreted factors and/or extracellular matrix–mediated effects—may also play important roles. Future experimental and theoretical approaches that independently manipulate tissue geometry, mechanical properties, and signaling dynamics will be required to clarify how these processes are integrated during mandibular morphogenesis.

Our findings raise several important questions for future investigation, particularly regarding how molecular and cellular mechanisms both contribute to, and operate within, the dynamically evolving tissue geometry described above. While we demonstrated the importance of Shh signaling in establishing cell polarity in the vOE, the detailed molecular mechanisms underlying this process remain unclear. We previously found that Shh signaling regulates planar cell polarity by controlling mechanosensation in neuroepithelial cells during early forebrain development, thereby allowing cells to determine their polarity based on principal stress directions (*11*). A similar mechanism may operate during mandibular morphogenesis. Elucidating the downstream molecular mechanisms of Shh signaling represents an important future direction. In particular, vOE-specific perturbation of Shh downstream effectors—once these mechanisms are clarified—would provide further support for our conclusion. If such perturbations were to phenocopy the effects observed following our vOE-specific inhibition of myosin activity, this would substantially strengthen the conclusion that vOE folding promotes the elongation and fusion of the mesenchymal mandibular primordia. Moreover, while polarized cell shape and signaling are widely observed throughout the vOE, prominent tissue folding and cell rearrangement occur specifically in the anterolateral region, whereas the anteromedial region undergoes pronounced isotropic tissue shrinkage driven by in-plane area reduction. These observations suggest that additional region-specific factors—potentially involving feedback between the mandibular mesenchymal primordia and the vOE—define the spatial patterning of distinct morphogenetic behaviors and that identification of molecular markers underlying such regional differences, potentially through future spatial transcriptomic analyses, may enable more targeted perturbations to further dissect these mechanisms.

The origin and broader roles of the OE in facial development also deserve further exploration. The OE contributes to PA1 aggregation (*29*, *43*), establishes facial D-V compartmentalization (Fig. 2A), and, as demonstrated in this study, drives mandibular formation through folding of the vOE (Fig. 4C). The dorsal OE also interacts with the forebrain to give rise to the pituitary primordium (*51*, *53*). Furthermore, in later stages, the vOE is known to play crucial roles in the mandibular skeletal growth/development (*22*, *27*, *34*), and through interactions with adjacent tissues, OE gives rise to the epithelial components of teeth and salivary glands, as well as taste buds (*54*). Collectively, these lines of evidence suggest that the OE has broader functions in craniofacial development than previously recognized. Despite arising from a common two-layered BE, the dorsal and ventral OE exhibit notable distinct morphogenetic behaviors. How such regional specialization emerges remains unresolved. To address this, spatial omic approaches may clarify how gene expression domains are regionalized within the BE and how they relate to OE positioning and functional diversification during development.

Last, our results may have evolutionary implications. The jaw is a hallmark of gnathostomes, and the requirement of vOE folding for mandibular formation suggests that OE-PA1 interactions may have contributed to jaw emergence. Comparing OE-PA1 interactions in jawless vertebrates like lampreys may provide important insights into the origins of jaw formation. Moreover, our findings suggest that epithelial folding patterns may more broadly contribute to the fundamental architecture of the entire face, beyond the mandible. Quantitatively analyzing epithelial tissue dynamics across the head region will be an important step toward understanding the physical mechanisms that underlie complex facial morphogenesis.

## MATERIALS AND METHODS

### Zebrafish husbandry and lines

Zebrafish (*Danio rerio*) adults and larvae were maintained at 28.5°C in a recirculating water system. The transgenic lines generated in this study included *Tg(EF1*α*:H2B-EGFP)*, *Tg(hsp:myh9b-*Δ*H-EGFP, ubi:NLS-tdTomato)*, and *Tg(hsp:EGFP, ubi:NLS-tdTomato)*. The transgenic lines produced in previous studies included *Tg(−4.9sox10:EGFP)^ba2^* (*55*), *Tg(hsp70l:mCherry-T2A-CreERT2)^tud104^* (*56*), and *Tg(ΔNp63:Gal4; UAS:Lifeact-GFP)* [comprising *TgBAC(ΔNp63:Gal4FF)^la213^* and *Tg(UAS:Lifeact-GFP)^mu271^*] (*57*, *58*). The homozygous *shhb* mutant line (*shhb^−/−^*) (*59*) was also used. The RIKEN WILD (RW) strain was used as the wild-type fish in this study. Stable transgenic lines were established by crossing to the RW strain, and the published lines were maintained by crossing to the RW strain. *shhb^−/−^* mutants were maintained by in-crossing. Embryos were maintained in E3 medium (*60*) at 28.5°C until 4 to 6 days postfertilization (dpf). The experiments were approved by the Ethics Committee of RIKEN Center for Biosystems Dynamics Research (approval no. A2024-06) and performed under the institutional ethical guidelines.

### Cloning of *myh9b*

Previously annotated isoforms of the *myh9b* cDNA in *D. rerio* (National Center for Biotechnology Information RefSeq: XM_001920063.7 and XM_005163701.4) encode truncated polypeptides that differ in their C-terminal regions compared with other MYH9 homologs, as noted by Gutzman *et al.* (*61*). To test whether a full-length Myh9b comparable to other MYH9 homologs exists, we cloned the 3′ region of the *myh9b* cDNA sequence. A cDNA library was synthesized from the total RNA of RW-strain embryos at 24 hpf using the PrimeScript II High Fidelity RT-PCR (reverse transcription polymerase chain reaction) Kit (TaKaRa) and the oligo dT primer adding adapter sequence (5′-CTGATCTAGAGGTACCGGATCCTTTTTTTTTTTTTTT-3′). The fragment of *myh9b* cDNA downstream region including 3′ untranslated region was amplified by PCR using a gene-specific primer (5′-CCCATCGATGCCACCGAGCAAAGAGAACGAGAAGAAG-3′) and a primer including the adapter sequence (5′-TTCTAGAGGCTCGAGCTGATCTAGAGGTACCGGATCC-3′). The amplified products that contained the flanking site for an In-Fusion reaction were fused to the pCS2+ vector linearized by PCR (5′-CTCGAGCCTCTAGAACTATAGTGAGTCG-3′ and 5′-GGTGGCATCGATGGGATCCTG-3′) using the In-Fusion HD Cloning Kit with Cloning Enhancer (TaKaRa, 639633). The inserts were verified by sequencing, which matches the current *myh9b* reference and is consistent with a recent report (*62*). The full-length *myh9b* coding sequence was cloned via PCR using the primer set (5′-GGAGTTCAACTACGAGTGGAAG-3′ and 5′-AGCGCTACGTTCATCCTTATAC-3′).

### Synthesis of mRNA

NLS-tdTomato, tdTomato-NLS, and zebrafish codon-optimized Tol2 transposase (zT2TP) mRNA (*63*) were synthesized from a pCS2+ plasmid containing the coding sequence linearized with NotI (TaKaRa, 1166A) using the mMESSAGE mMACHINE SP6 Transcription Kit (Thermo Fisher Scientific, AM1340). Synthesized RNA solution was purified using the MEGAclear Transcription Clean-Up Kit (Thermo Fisher Scientific, AM1908) or the PureLink RNA Mini Kit (Thermo Fisher Scientific, 12183020). The properly diluted RNA solution or mixture was injected into one-cell stage embryos.

### Generation of transgenic lines

The full-length coding sequence of the cloned *myh9b* was fused to an EGFP coding sequence via a linker sequence encoding DPPVAT peptides by assembling three DNA fragments amplified by PCR. The primers used were as follows: the *myh9b* coding sequence with linker peptides (5′-ATCGATGCCGCCACCATGTCGGACGTGGACAAG-3′and 5′-GGTGGCGACCGGTGGATC-3′), the EGFP coding sequence (5′-GGTGGCGGCATCGATGGGATCCTGCAAAAAGAAC-3′and 5′-CCACCGGTCGCCACCATGGTGAGCAAGGGCGAGGA-3′) from T2AL200R150G (*64*), and the linearized pCS2+ vector (5′-CCACCGGTCGCCACCATGGTGAGCAAGGGCGAG-3′and 5′-TTCTAGAGGCTCGAGTTACTTGTACAGCTCGTCCATGC-3′). A head domain–deleted (ΔH) myosin heavy chain, lacking the adenosine triphosphatase (motor) domain, acts as a dominant-negative myosin (*65*, *66*). We therefore introduced a head domain deletion into *myh9b* (*myh9b-ΔH*) by site-directed mutagenesis of pCS2+ myh9b-EGFP using the PrimeSTAR mutagenesis kit (TaKaRa, R046A) with a set of primers for the mutation (5′-CACCATGAGACAGCAGCAGCTGACA-3′and 5′-TGCTGTCTCATGGTGGCGGCATCGAT-3′). The hsp (heat shock element based) flanked by insulators on each side, derived from the plasmid pPBIS19-*mgfc*:TagBFP-8x*HSE:Cre* (*67*), was assembled such that the target coding sequences (i.e., Myh9b-ΔH-EGFP or EGFP) were placed immediately downstream of the hsp cassette. The *ubi:NLS-tdTomato* fragment was integrated in tandem into this construct. The entire assembled sequence was inserted between the 200-bp left and 150-bp right Tol2 arms (*64*). The construct to establish the *Tg(EF1*α*:H2B-EGFP)* line was generated by sequentially linking the EF1α promoter, the histone H2B coding sequence, the linker sequence encoding DPPVAT, and the EGFP sequence, in that order. Twenty-seven picograms of the established plasmid was injected with 35 pg of zT2TP mRNA in 86 mM KCl, following the established protocols (*63*, *68*). We identified founders for *Tg(hsp:myh9b-ΔH-EGFP, ubi:NLS-tdTomato)* and *Tg(hsp:EGFP, ubi:NLS-tdTomato)* by screening F1 offspring for heat shock–induced fluorescence and established stable lines. For whole-mount heat shock, the tube block of the block incubator (Astec, BI-526T) was removed, and the chamber was filled with water. The temperature of the incubator was set to 39°C, and a 35-mm dish (IWAKI, 1000-035) that contained embryos was immersed in the heated water for 1.5 hours. After heating, the dish was transferred to and maintained at 28.5°C, and the embryos were observed under a fluorescence stereomicroscope to check for induced fluorescent signals, which were detectable within 6 hours after heating.

### Time-lapse imaging

3D time-lapse imaging of live embryos was performed using an upright multiphoton microscope (FVMPE-RS, Evident Scientific). For time-lapse imaging of fluorescently labeled NCCs, embryos were obtained by crossing *Tg*(*sox10:EGFP*) with the RW strain. The obtained embryos were injected with NLS-tdTomato or tdTomato-NLS mRNA at the one-cell stage for detection of nuclei. The dechorionated embryo at the target stage was embedded in 0.6% low-melting point (LMP) agarose (Lonza, 50101) in E3 medium with 0.002% PTU (*N*-phenylthiourea; Sigma-Aldrich, P7629) and tricaine (0.72 mg/ml; ethyl 3-aminobenzoate methanesulfonate; Merck, A5040). Agarose gel around the head was carefully removed using a glass capillary needle. Mounted embryos were cultured in E3 medium with 0.002% PTU and tricaine (0.72 mg/ml) and incubated in a stage-top incubator (TOKAI-Hit) at ~28.5°C during imaging. For time-lapse imaging with pharmacological treatments, embryos were mounted in mounting gel and medium, both containing either 100 μM cyclopamine (LKT Laboratories; dissolved in ethanol) for Shh inhibition or 20 μM (-)-blebbistatin [Cayman Chemical Company; dissolved in dimethyl sulfoxide (DMSO)] for myosin inhibition. Images were acquired every 30 min at 1.2- or 1.5-μm intervals for more than 12 hours using a 25×/1.05 numerical aperture objective (XLPLN25XWMP, Evident Scientific) with excitation wavelengths of 900 and 1040 or 1045 nm. To avoid the phototoxicity of (-)-blebbistatin at certain GFP excitation wavelengths [ranging from 450 to 490 nm (*69*)], the time-lapse imaging of embryos treated with (-)-blebbistatin was performed using only the 1045-nm laser. Cell lineages in time-lapse imaging data were determined by tracing cells using the TrackMate plugin in Fiji.

### Pharmacological treatment

Dechorionated embryos were placed into 2 ml of E3 medium containing 0.002% PTU in a glass dish. For inhibition of cell division, 100 μM aphidicolin (FUJIFILM; dissolved in DMSO), or 1% DMSO as a control, was added to the medium from 34 or 72 hpf onward. For inhibition of myosin activity, 20 μM (-)-blebbistatin, or 0.2% DMSO as a control, was added to the medium from 35 hpf onward. For inhibition of Shh signaling activity, 100 μM cyclopamine, or 1% ethanol as a control, was added to the medium from 24 hpf onward.

### Head removal by surgical dissection

In two sets of experiments—one aiming to obtain clear dorsal views of the OE sheet or mandibular mesenchyme and the other to assess the influence of the dorsal head region on mandibular morphogenesis—the head region, including the brain and eyes, was surgically removed. Embryos at 36 or 48 hpf were embedded in a 0.6% LMP agarose gel of 1× low calcium-magnesium modified Ringer’s (LCMR) (pH 7.6) (*70*) or modified Tyrode’s solution [150 mM NaCl, 4 mM KCl, 1.5 mM CaCl_2_, 1 mM MgCl_2_, and 5 mM Hepes (pH 7.6)], hereafter referred to as glucose-free Tyrode’s solution (GFTS), containing 0.002% PTU and tricaine (0.72 mg/ml). After the gel was solidified enough, the GFTS containing 0.002% PTU and tricaine (0.72 mg/ml) was added. The head region of embryos was dissected using a Gastromaster (Xenotek Engineering) with an electrode (Protech, 13-Y1). The head-removed samples were removed from the gel using forceps and immediately fixed using a solution containing 4% paraformaldehyde (PFA), 40 mM Na_2_PO_4_, and 10 mM NaH_2_PO_4_ (4% PFA in phosphate buffer) for 2 to 4 hours at room temperature (RT). For detection of the phosphorylated myosin light chain (pMLC), PhosSTOP (one tablet for 10 ml; Roche, 04906845001) was added to 4% PFA in phosphate buffer. After fixation, the samples were either washed with PBS-T [phosphate buffer saline (NIPPON GENE, 314-90185) with 0.1% Tween 20 (FUJIFILM, 166-21213)] for immunostaining or with methanol for in situ hybridization. To assess the effect of the dorsal head region on mandibular morphogenesis, the dorsal head of embryos injected with tdTomato-NLS mRNA at the one-cell stage was surgically removed at 36 hpf. Following removal, the samples were maintained in GFTS containing 0.5 mM glucose, gentamicin sulfate solution (5 μg/ml; NACALAI TESQUE), and 0.002% PTU. Head-removed samples were fixed at 48 hpf using 4% PFA in phosphate buffer for 3 hours at RT, rinsed with PBS-T, and imaged using a 30× silicon immersion objective lens (UPLSAPO30XS, Evident Scientific) on a confocal microscope (FV3000, Evident Scientific) with z-stack intervals of 2 μm. Intact embryos were imaged at 48 hpf using a 25× water-immersion objective lens (XLPLN25XWMP, Evident Scientific) on a multiphoton microscope (FV4000, Evident Scientific), also with z-stack intervals of 2 μm. Samples were additionally fixed at 4 dpf in 4% PFA in phosphate buffer for 3 hours at RT.

### Immunohistochemistry

Embryos were fixed at the target stage using 4% PFA in phosphate buffer for 2 to 4 hours at RT. After fixation, embryos were washed with PBS-T two or three times and stored at 4°C. The fixed samples were permeabilized using PBS containing 2% Triton X-100 (Wako, 162-24755) (2% Triton X-100/PBS solution) for 1.5 hours at RT and then rinsed with PBS-T two or three times. The samples were blocked with blocking solution [PBS-T containing 1% bovine serum albumin (Sigma-Aldrich, A7888) and 2% goat serum (Thermo Fisher Scientific, 16210064)] for 1 hour at RT. The samples were incubated with one of the following primary antibodies in the blocking solution for one to two nights at RT: anti-pMLC (1:20; Cell Signaling Technology, 3671), anti-phosphorylated histone H3 (pH3; 1:200; Millipore, 06-570), or anti-laminin (1:200; Sigma-Aldrich, L9393). In addition, anti-GFP (1:300; NACALAI TESQUE, 04404-84) was used either alone or coincubated with the above primary antibodies, as required. After the incubation, the samples were washed with PBS-T six times in 3 hours, then were blocked with the blocking solution, and incubated with a secondary antibody—Alexa Fluor 488–, 568–, or 647–conjugated immunoglobulin G antibody (Thermo Fisher Scientific)—in the blocking solution overnight at RT. Hoechst33342 (5 μg/ml; Thermo Fisher Scientific, H3570) and Alexa Fluor 546– or 568–conjugated phalloidin (1:100; Thermo Fisher Scientific) were added to the primary antibody solution and the secondary antibody solution when necessary for nuclei staining or visualization of cellular membranes. The stained samples were stored in the secondary antibody solution at 4°C and washed with PBS-T several times before confocal imaging.

### In situ hybridization chain reaction

Embryos were fixed at the target stage using 4% PFA in phosphate buffer for 2 to 4 hours at RT. After fixation, embryos were washed with methanol two or three times and stored in methanol at −30°C. In situ hybridization chain reaction (HCR) was performed according to the manufacturer’s protocol provided by Nepa Gene, with a modification in which the samples were permeabilized by 2% Triton X-100/PBS solution for 1.5 hours at RT after the rehydration and before the prehybridization step. For fluorescent labeling of target mRNAs, either ISHpalette Short hairpin amplifier ATTO647N-A161 or Cyanine5-A161 (Nepa Gene, IPL-B-A161) was used. After HCR, immunostaining for the detection of GFP, pH3, and/or nuclei was performed using anti-GFP antibody, anti-pH3 antibody, and Hoechst33342, respectively. Since the blocking solution dispersed and weakened the HCR fluorescent signals, the samples were incubated in PBS-T only. The sets of oligo probes for mRNA are shown in table S1.

### Confocal imaging

Confocal images of the immunostained or HCR-labeled samples were acquired using the 10× (UPLXAPO10X, Evident Scientific), 20× (UPLXAPO20X, Evident Scientific), or 30× silicon-immersion (UPLSAPO30XS, Evident Scientific) objective lenses on FV3000 or FV4000 confocal microscopes (Evident Scientific). Samples were mounted in 0.6% LMP agarose in E3 medium on a 35-mm glass-bottom dish. For the detection of pMLC localization, *ptch2* expression in the OE, analysis of cell division, and analysis of cell shapes (stained with phalloidin), samples were imaged using a 30× silicon-immersion objective lens with z-stack intervals of 0.8 or 1.6 μm. For the detection of *shha* expression in the entire head, samples were imaged in the lateral view using the 10× objective lens with 4-μm z-stack intervals and from the anterior side using the 20× objective lens with 1.0-μm z-stack intervals. For the observation of mandibular arch elongation and vOE folding at 48 or 50 hpf, samples were imaged using the 30× silicon-immersion objective lens with 1.2-μm z-stack intervals. To visualize the spatial pattern of *shha* mRNA localization within internal embryonic tissues, we generated 3D surface masks in Imaris and extracted fluorescence within a subvolume that encompassed the ventral diencephalon, the mandibular mesenchyme, the OE, and the pharyngeal endoderm at 28 and 32 hpf.

### Image alignment for quantitative orientation analysis

To analyze orientational features such as cell division axis angles, cell shape anisotropy, and the polarity of pMLC localization, imaging data were rotated before quantification. First, the z-stack imaging data were corrected through 3D rotation using Imaris so that the vOE facing the oral cavity was oriented parallel to the *XY* plane. After this 3D rotation, the midline of each embryo was defined on the basis of anatomical landmarks (e.g., the notochord center or positions of paired pharyngeal arches). The imaging data were then further rotated in two dimensions using Fiji to align the midline with the *Y* axis.

### Surface reconstruction of 3D organ and tissue polygons

3D polygonal surface models representing organ or tissue morphology were reconstructed from multiphoton live imaging data using Imaris or Fiji. Closed surface models of the eyes, lenses, brain, PA1, and periderm were generated in Imaris by connecting manually drawn outlines. The BE and OE were reconstructed as open surfaces based on their contours. Outlines of the basal side of the OE and its continuous BE were manually drawn in Fiji using the “Freehand Line” tool from frontal or lateral views. Triangular meshes were constructed by generating edges between adjacent vertices along the outlines. Mesh groups representing structures intended to be connected but were separated were manually unified to create a continuous surface. The resulting 3D polygonal models were refined by adjusting mesh density and applying surface smoothing using MeshLab.

### Extraction and projection of ventral oral ectodermal voxel data

To analyze the cell shape anisotropy and pMLC localization in vOE cells, voxels corresponding to the vOE were extracted from imaging data. An open-surface polygon representing the vOE was reconstructed as a polygon based on manually drawn outlines. Because the OE consists of a thin bilayer in which the ventral and dorsal epithelial sheets face each other at their apical surfaces, voxels from the dorsal sheet could potentially contaminate the data. To avoid this, outlines were traced along the more basal side of the vOE. Cell shapes were manually delineated in Fiji using the Freehand Line tool. The resulting 3D polygon was further refined by adjusting the mesh density and applying surface smoothing using MeshLab. Next, we calculated a set of normal vectors (0.5 μm in length) originating from the centroid of each triangular mesh composing the vOE. Voxels intersecting these vectors were extracted. After rotating the imaging data (see the “Image alignment for quantitative orientation analysis” section), the extracted vOE voxel data were projected onto the *XY* plane as a maximum intensity projection.

### Evaluation of cellular rearrangements within the vOE

Cellular rearrangements within the vOE were assessed using time-lapse imaging data. A rearrangement event was defined as the loss of adjacency between two cell lineages that were adjacent at the initial time point but no longer in contact at the final time point. When a cell divided during the observation period, its progeny were treated as part of the parent lineage for adjacency analysis.

### Analysis of cell division and cell shape orientations

To examine the contributions of oriented cell division and/or cell shape changes to the elongation of the mandibular mesenchyme, we quantified their 2D orientation angles in the coronal section where the cross-sectional area of the mandibular mesenchyme was maximal. As described in the “Image alignment for quantitative orientation analysis” section, the image stacks were rotated so that the vOE was aligned parallel to the *XY* plane, and the midline aligned with the *Y* axis. For analyzing cell division orientation, we identified two chromosome centers during anaphase or telophase in each of the *sox10* reporter–labeled cells (i.e., NCCs within the presumptive Meckel’s region). The orientation angle was then measured by projecting the line connecting the two chromosome centers onto the coronal plane and calculating its angle relative to the M-L axis. Cell shapes were identified by manually tracing the outlines of cell membranes labeled with phalloidin in an optical slice corresponding to the coronal section described above. The orientation angle of each cell was then measured as the angle between its longitudinal axis and that of the mandibular mesenchymal cell population, defined as the direction of maximum spatial variance in cell positions, which closely approximates the direction of tissue elongation.

### IR irradiation for the local induction of target genes

Local heat shock stimulation was applied using the IR-LEGO system (*50*) to embryos of either *Tg(hsp:myh9b-*Δ*H-EGFP, ubi:NLS-tdTomato)* or *Tg(hsp:EGFP, ubi:NLS-tdTomato)* at 24 to 30 hpf. Because *Tg(hsp:EGFP, ubi:NLS-tdTomato*) embryos showed weak nuclear fluorescence, NLS-tdTomato mRNA was injected at the one-cell stage to enhance nuclear labeling. To achieve strong induction of the dominant-negative form of myosin in response to heat shock, embryos were generated by crossing two independent stable lines of *Tg(hsp:myh9b-ΔH-EGFP, ubi:NLS-tdTomato)*. Dechorionated embryos were mounted in 0.6% LMP agarose in E3 medium containing 0.002% PTU and tricaine (0.72 mg/ml), on a 35-mm glass-bottom dish (IWAKI, 3971-035). To minimize the distance between the OE and the objective lens, samples were tilted and positioned in direct contact with the glass surface of the glass-bottom dish during mounting. Infrared (IR) laser irradiation was applied from two directions, the front and the lateral side. The IR laser system used in this study consisted of three components: a Fabry-Pérot laser emitting at 1475 nm (FiberLabs, FPLD-1475-490-CP), a shutter controller (SIGMA KOKI, SSH-C2B), and a stage controller (SIGMA KOKI, GIP101). The system was connected to a microscope (Evident Scientific, IX83) equipped with a CSU-W1 confocal scanning unit (Yokogawa) and an iXon electron-multiplying charge-coupled device (EMCCD) camera (Andor, DU-888 U3-CS0-#BV). Sample observation and IR laser irradiation were performed using a 40× objective lens (UPLSAPO40X2, Evident Scientific). The power of the IR laser passing through the objective lens and glass-bottom dish was measured with a thermal detector (gentec-EO, UP17P-6S-H5-D0). IR laser irradiation was applied to cells in the vOE or BE near the already invaginated vOE, as identified in confocal images. Laser power and exposure time were adjusted according to the depth from the embryo surface: 19 mW for 7 s at 40-μm depth, 19 mW for 6 s at 30-μm depth, and 18 mW for 5 s at shallower depths. After IR laser irradiation, embryos were removed from the gel and maintained in E3 medium containing 0.002% PTU at 28.5°C. At 48 hpf, embryos were fixed in 4% PFA in phosphate buffer for 2 to 3 hours at RT. Because the IR laser traversed the periderm overlying the target tissues, gene expression was also induced in the periderm. However, we confirmed that this peridermal induction had no effect on either the folding of the vOE or elongation/fusion of the mandibular mesenchyme. To avoid nonspecific fluorescence signals, including those from the periderm and from regions of the BE that were not adjacent to the vOE, we used Imaris to mask and extract fluorescence signals specifically in the vOE, the surrounding BE, and the mandibular mesenchyme (Fig. 4C). Some live embryos exhibiting the unfused mandibular mesenchyme phenotype were maintained and imaged at 53 hpf using the 30× silicon-immersion objective lens on the FV3000 confocal microscope and subsequently fixed at 4 dpf in 4% PFA in phosphate buffer for 2 to 3 hours at RT.

### Ablation of PA1 by IR irradiation

PA1 ablation was performed by IR laser irradiation. Dechorionated *Tg(sox10:EGFP)* embryos injected with tdTomato-NLS mRNA at the one-cell stage were mounted in 0.6% LMP agarose in E3 medium supplemented with 0.002% PTU and tricaine (0.72 mg/ml), on a 35-mm glass-bottom dish. Imaging and IR laser irradiation were conducted from the lateral side of embryos at 32 hpf using a 40× objective lens (UPLSAPO40X2, Evident Scientific). IR laser pulses (18 mW, 5 s per pulse) were repeatedly applied to neural crest–derived cells in the mandibular primordium, as identified in confocal images, until cell ablation was achieved. This procedure targeted the entire mandibular mesenchyme. After laser ablation, embryos were removed from the gel and incubated in E3 medium containing 0.002% PTU at 28.5°C. Embryos were fixed at 4 dpf in 4% PFA in phosphate buffer for 2 to 3 hours at RT.

### Alcian blue staining

Larvae at 4 or 5 dpf were fixed in 4% PFA in phosphate buffer for 3 hours at RT and washed several times in PBS-T. After washing, samples were dehydrated by replacing the buffer with 70% ethanol for at least 1 hour. Staining was performed by incubating the samples overnight in Alcian blue solution [Alcian Blue 8GX (0.1 mg/ml; Sigma-Aldrich, A5268) in 80% ethanol and 20% acetic acid]. After staining, samples were washed in PBS-T, and images were acquired using a stereo microscope (Leica, M125 C).

### Quantification of polarity patterns in cell shape and pMLC localization in the vOE

Images of the vOE at 36 hpf were used for the quantitative analysis of polarity patterns in cell shape and pMLC localization. These images were generated by projecting the extracted voxels corresponding to the vOE onto the *XY* plane (see the “Extraction and projection of ventral oral ectodermal voxel data” section). Cell edges were initially detected using the standard watershed algorithm in Fiji, followed by manual correction based on F-actin staining or Lifeact-GFP localization. To ensure reliable orientation measurements, very short edges (<3 μm or <10 pixels) were excluded from analysis. For each segmented cell, an ellipse-fitting procedure was applied to determine the orientation of the long axis and the centroid position. pMLC intensity at each cell edge was defined as the mean intensity of pixels within a 1-pixel-dilated region surrounding the detected edge. For each cell, the edge with the highest pMLC intensity was taken to represent the orientation of pMLC localization polarity. To determine the center of the concentric pattern of cell shape and pMLC orientations, we first assumed that the center lies along the midline. A candidate point on the midline was selected and used as a provisional center. For each cell, we computed the angle between the radial axis (connecting the cell centroid to the candidate center) and the cell’s long axis. The distribution of these angles across all cells was approximated by a von Mises distribution, and the concentration parameter κ was computed. This calculation was repeated for each point along the midline, and the point yielding the maximum κ value was defined as the center of the concentric pattern. Using this center, we also quantified the degree to which pMLC localization polarity followed a concentric orientation pattern.

### Analysis of cell shape and area in the vOE

To examine the contribution of cell shape changes in the anterolateral (R1) region of the vOE to its lateral folding, we quantified cell shapes in R1 at 48 hpf. The optical plane intersecting the folded R1 region was determined from 3D confocal imaging data of immunostained embryos at 48 hpf. Cell boundaries were initially segmented using the standard watershed algorithm in Fiji, followed by manual correction based on F-actin staining or Lifeact-GFP localization. The axis of tissue elongation was estimated from the position of landmark cells inferred from time-lapse imaging data. In-plane cell area—defined as the cross-sectional area parallel to the epithelial sheet—was measured in phalloidin-stained embryos at 36 and 48 hpf.

### Quantitative analysis of tissue and cell dynamics in PA1

To quantify tissue-level deformation in the PA1, we extracted trajectories of individual cells from time-lapse imaging data and reconstructed a 3D deformation map (*x*, *y*, *z*) = **φ**(*X*, *Y*, *Z*) = [φ_1_(*X*, *Y*, *Z*), φ_2_(*X*, *Y*, *Z*), φ_3_(*X*, *Y*, *Z*)] from 36 to 48 hpf using Gaussian process regression. The regression was implemented in Python 3.12.7 using GPy (version 1.13.2). The uppercase coordinates (*X*, *Y*, *Z*) represent positions within PA1 at 36 hpf (before deformation), and the lowercase coordinates (*x*, *y*, *z*) represent positions at 48 hpf, when the fusion of bilateral mandibular primordia occurs at the midline. Because statistical regression methods tend to show increased estimation error near domain boundaries, we augmented the dataset by orthogonally projecting data points located near the PA1 boundary onto the boundary surface. These projected points were included in the regression process to improve accuracy near the boundary. The validity of this method was assessed by cross-validation based on prediction error. For three independent embryos, the prediction errors were ~5 μm, indicating that the resulting deformation map adequately captured the observed data and provided sufficient predictive accuracy. From the obtained deformation map **φ**, we calculated the deformation gradient tensor **F** (= d**φ**/d**X**), the right Cauchy-Green deformation tensor **C** (= **F**^T^**F**), the stretch tensor **U** (defined by **C** = **U**^2^), and the rotation tensor **R** (from the polar decomposition **F** = **RU**). On the basis of these, we computed the volume growth rate (det**F**; color dots in Fig. 1E, left), the magnitude of deformation anisotropy ($1-\sqrt{\lambda_{2}\lambda_{3}}/\lambda_{1}$), and the orientation of anisotropy ($\nu_{1}$; line segments in Fig. 1E), where $\lambda_{i}$ ($\lambda_{1}>\lambda_{2}>\lambda_{3}$) and $\nu_{i}$ are the eigenvalues and eigenvectors of **U**. Principal components analysis of deformation anisotropy was conducted on the basis of the eigenvalues and eigenvectors of **U** at each point (Fig. 1F). In Fig. 1H, cell rearrangement was visualized as follows. First, the inverse of the rotation tensor R was applied to the cell position vectors at 48 hpf to display them in the same coordinate frame as the cell positions at 36 hpf. The cell positions at 36 and 48 hpf were then projected onto a 2D plane spanned by the directions of maximal and minimal deformations, allowing relative positional changes between neighboring cells to be more easily visualized.

### Quantitative analysis of tissue and cell dynamics in the vOE

As described in the Results, the vOE was divided into four regions by bisecting it along the M-L and A-P axes (Fig. 2D). Since each region could be approximated as a nearly planar surface, we independently estimated a representative plane for the cell distribution at 36 hpf and at 48 hpf, respectively, by fitting a plane such that its normal vector aligned with the direction of minimal spatial variance in cell positions. Cell positions were then projected onto these planes. On the basis of the projected cell positions, tissue deformation was approximated using a 2D linear transformation L between the positions at 36 and 48 hpf. Because this linear transformation corresponds to the deformation gradient tensor for each region, we used it to compute two quantities characterizing the tissue deformation: the area growth rate and the magnitude of deformation anisotropy (Fig. 2E). To visualize cell rearrangement in region 1 (the anterolateral portion of the vOE), we applied the inverse of the rotation tensor to the cell positions at 48 hpf and displayed the result in the same coordinate frame as the positions at 36 hpf, as performed in the deformation analysis of PA1 (Fig. 3D).
