# Supplementary material for: A cross-tissue physicochemical causal chain underlying vertebrate mandibular morphogenesis
Source: Sci Adv. 2026 May 8;12(19):eaec7997. doi: 10.1126/sciadv.aec7997 (PMC13155309; doi:10.1126/sciadv.aec7997)
Supplement: Supplementary file 1 — Figs. S1 to S7 Table S1 Legends for movies S1 to S3 [file sciadv.aec7997_sm.pdf]

Science Advances
AAAS

Supplementary Materials for

A cross-tissue physicochemical causal chain underlying vertebrate mandibular morphogenesis

Kazutaka Hosoda *et al.*

Corresponding author: Yoshihiro Morishita, yoshihiro.morishita@riken.jp

Sci. Adv. **12**, eaec7997 (2026)
DOI: 10.1126/sciadv.aec7997

The PDF file includes:

Figs. S1 to S7
Table S1
Legends for movies S1 to S3

Other Supplementary Material for this manuscript includes the following:

Movies S1 to S3

Figure S1
Hosoda et al.

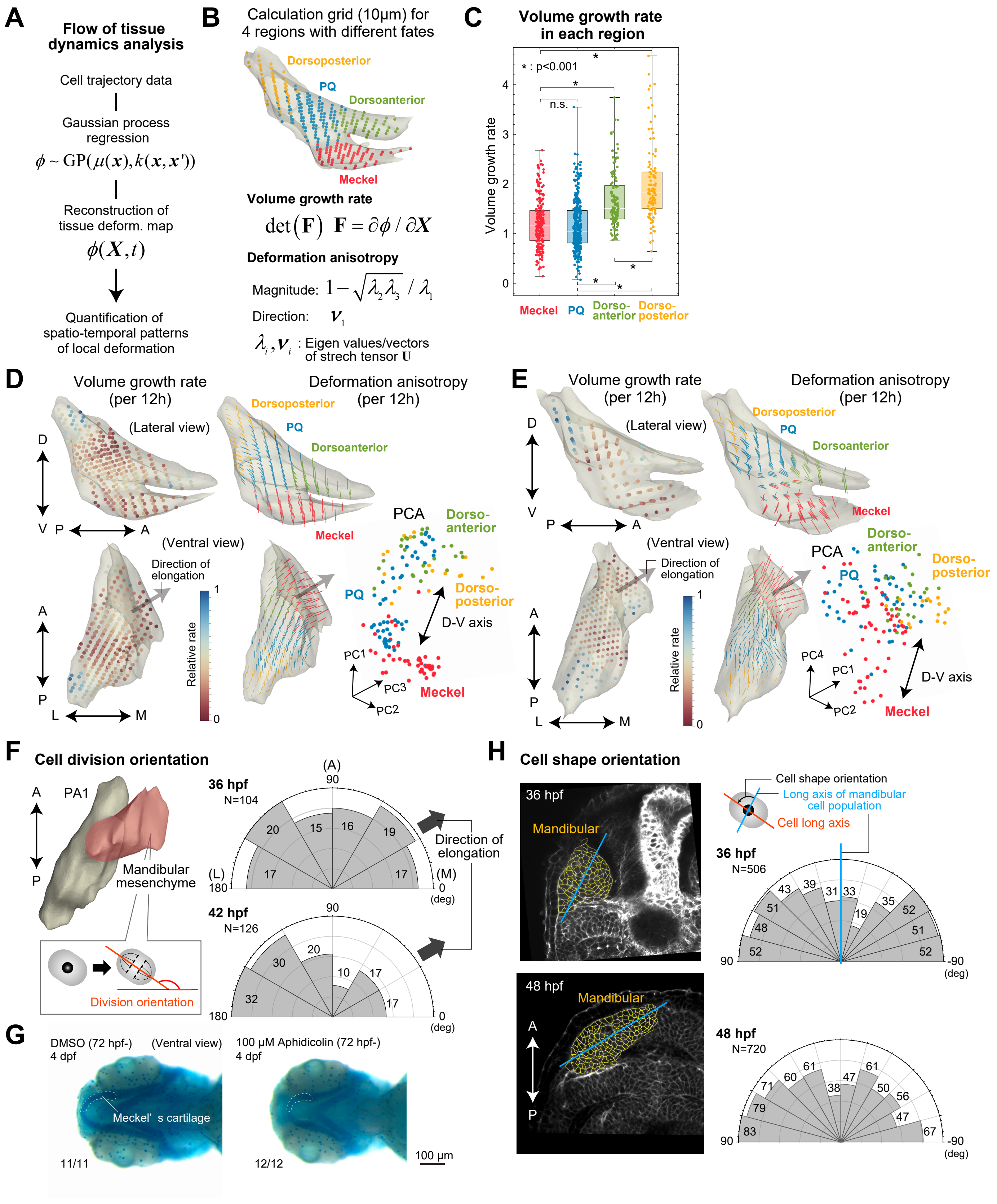

Fig. S1. Quantitative analysis of tissue/cell dynamics during jaw development.

(**A**) Workflow for analyzing tissue deformation dynamics based on the first pharyngeal arch (PA1) cell trajectory data.
(**B**) Grid points within the PA1 at 36 hours post-fertilization (hpf), where tissue deformation characteristics (volume growth rate and deformation anisotropy) were calculated. Grid points in four distinct regions within the PA1 are shown in different colors.
(**C**) Comparison of volume growth rate among the four regions of PA1. The volume growth rate was significantly lower in the presumptive Meckel's and palatoquadrate cartilage regions compared to the surrounding areas. Data represent combined results from three embryos. Box-and-whisker plots show the median (center line) and minimum and maximum values (whiskers). Statistical analysis was performed using a Student's *t*-test. n.s., not significant.
(**D, E**) The same analyses as shown in Fig. 1E and F were performed using data from two additional individuals, yielding similar results.
(**F**) Quantitative analysis of cell division orientation in mandibular neural crest-derivative cells (NCCs). The angular distribution of division orientations was plotted using a half-polar plot with six bins (30° per bin). The direction of mandibular primordium elongation is indicated by an arrow. We analyzed a total of 104 pairs of divided cells in 27 mandibular primordia at 36 hpf, and 126 pairs in 32 primordia at 42 hpf. L, lateral (180°); A, anterior (90°); M, medial (0°).
(**G**) Inhibition of cell proliferation at later stages reduced the size of Meckel's cartilage.
(**H**) Quantitative analysis of cell shape orientation in mandibular NCCs. (Left) Coronal optical sections of phalloidin-stained embryos at 36 hpf and 48 hpf. The shapes of mandibular NCCs are outlined in yellow. The longitudinal axis of mandibular NCC population, consistent with the direction of elongation, is indicated by cyan lines. A, anterior; P, posterior. (Right) Angular distributions of cell shape orientation at 36 hpf and 48 hpf. Angles relative to the longitudinal axis were plotted using a half-polar plot with 12 bins (15° per bin). We analyzed a total of 506 cells from five mandibular primordia at 36 hpf and 720 cells from five mandibular primordia at 48 hpf.

Figure S2
Hosoda et al.

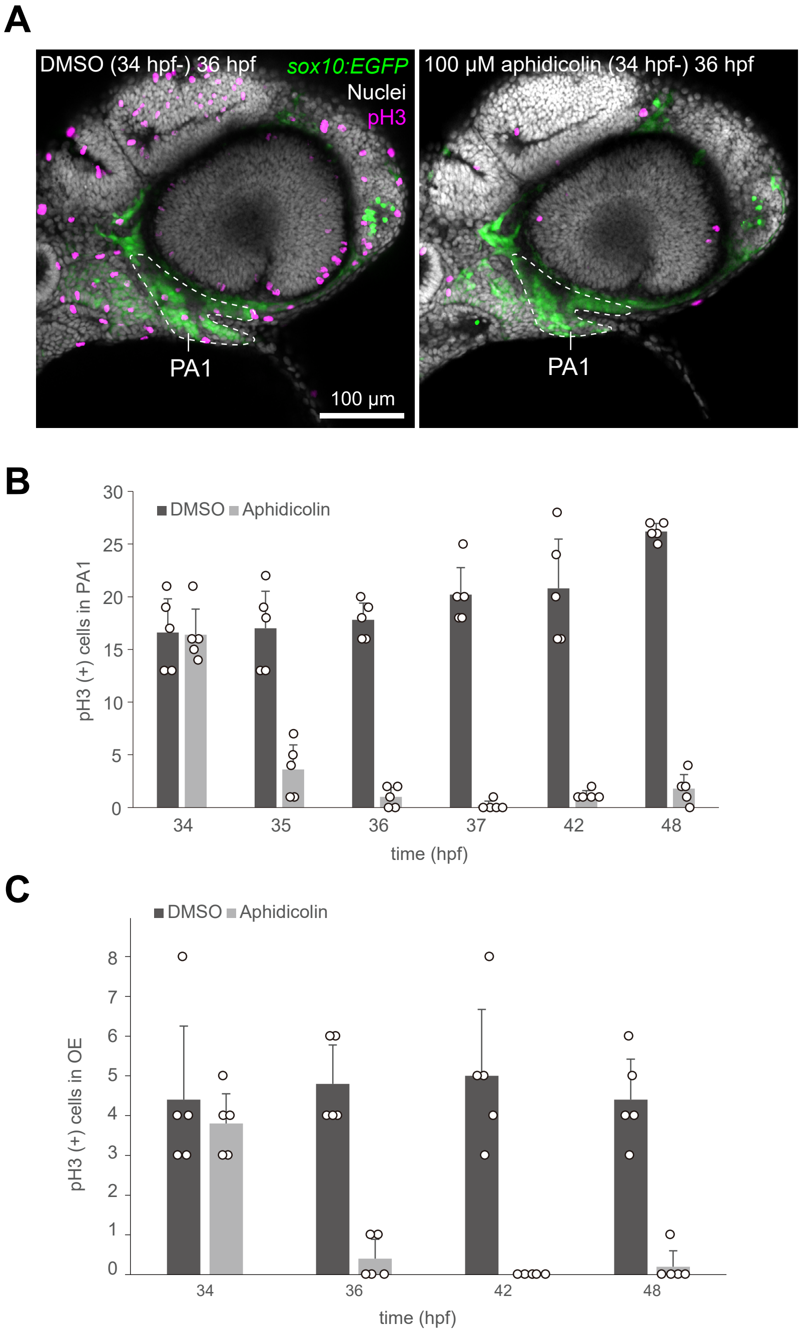

Fig. S2. Cell division is markedly inhibited by aphidicolin treatment.

(A) Optical sagittal sections of embryos at 36 hours post-fertilization (hpf) treated with DMSO (control, left) or 100 μM aphidicolin (right) from 34 hpf. Cells positive for phospho-histone H3 (pH3; a mitotic marker, magenta) are markedly reduced upon aphidicolin treatment. Nuclei detected by Hoechst 33342 are shown in white, and neural crest–derived cells are labeled by green fluorescence in *Tg(sox10:EGFP)* embryos. The first pharyngeal arch (PA1) is outlined by dashed lines. Anterior is to the right.

(B) Quantification of pH3-positive cells within the PA1 region of control and aphidicolin-treated embryos. All embryos were treated with DMSO or 100 μM aphidicolin from 34 hpf. Bars indicate mean values (n = 5 embryos); error bars indicate standard deviation.

(C) Quantification of pH3-positive cells within the oral ectoderm (OE) region of control and aphidicolin-treated embryos. All embryos were treated with DMSO or 100 μM aphidicolin from 34 hpf. Bars indicate mean values (n = 5 embryos); error bars indicate standard deviation.

Figure S3
Hosoda et al.

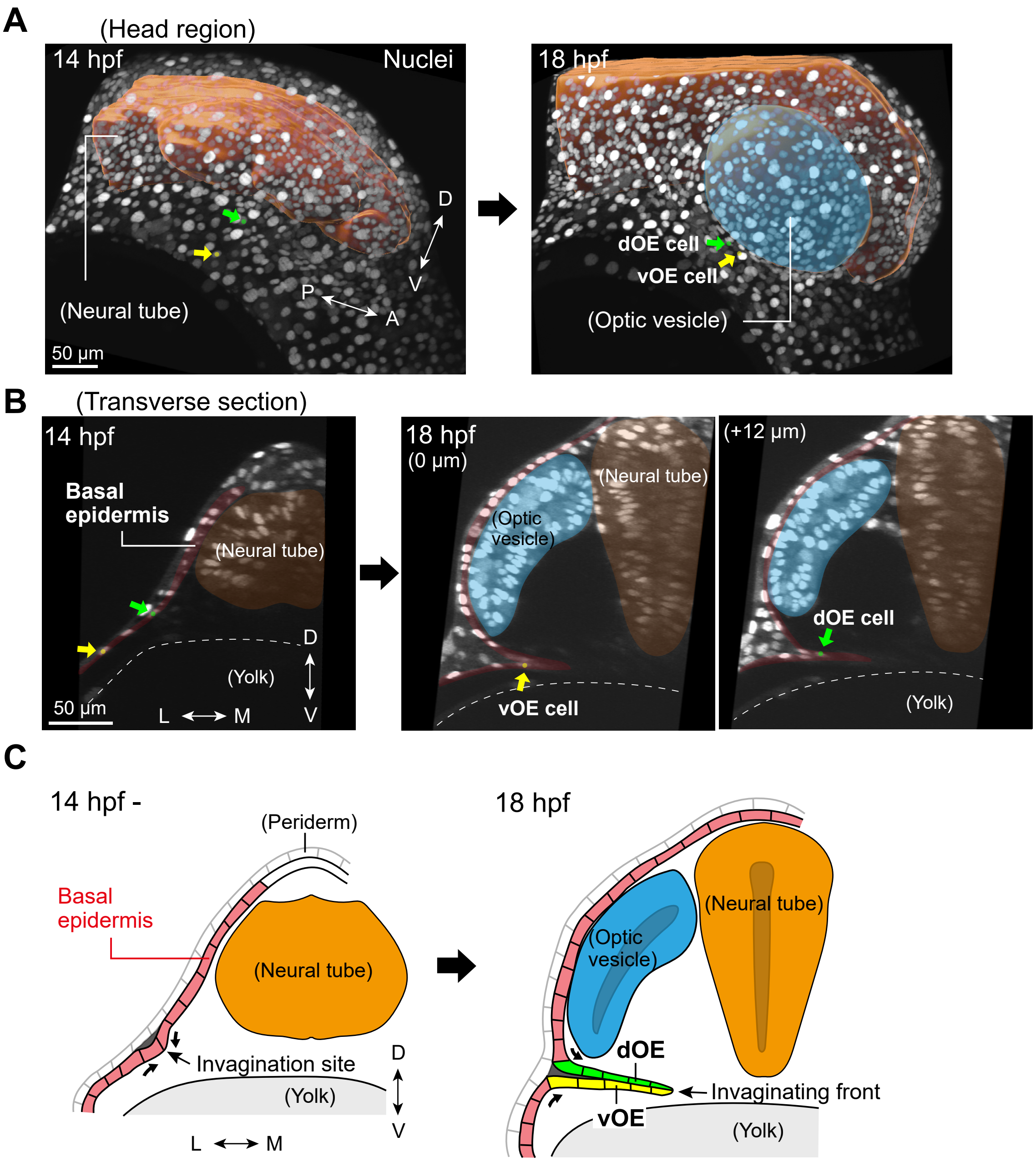

Fig. S3. Oral ectoderm is formed by invagination of the facial basal epidermis.

(A) Live imaging of a zebrafish embryo at 14 hours post-fertilization (hpf) (left) and 18 hpf (right). Nuclei (white) were visualized by tdTomato-NLS mRNA injection at the one-cell stage. The neural tube is shown as an orange polygon. By 18 hpf, the optic vesicle becomes evident (cyan). Representative tracked cells that eventually give rise to the dorsal oral ectoderm (dOE, green) and the ventral oral ectoderm (vOE, yellow) are indicated by dots and arrows.
(B) Optical transverse sections of the head region shown in (A). Images at 18 hpf are shown at two different anterior–posterior (A–P) levels; the right image is located 12 μm posterior to the left. Presumptive dOE (green) and vOE (yellow) cells at 14 hpf, which are located in the facial basal epidermis (red), move inward into the head region via invagination by 18 hpf, resulting in the formation of a bi-layered oral ectoderm (red).
(C) Schematic illustrating the invagination process of the facial basal epidermis leading to oral ectoderm formation, shown in the transverse plane. The anterior–posterior (A–P), dorsal–ventral (D–V), and medial–lateral (M–L) axes are indicated by arrows.

Figure S4
Hosoda et al.

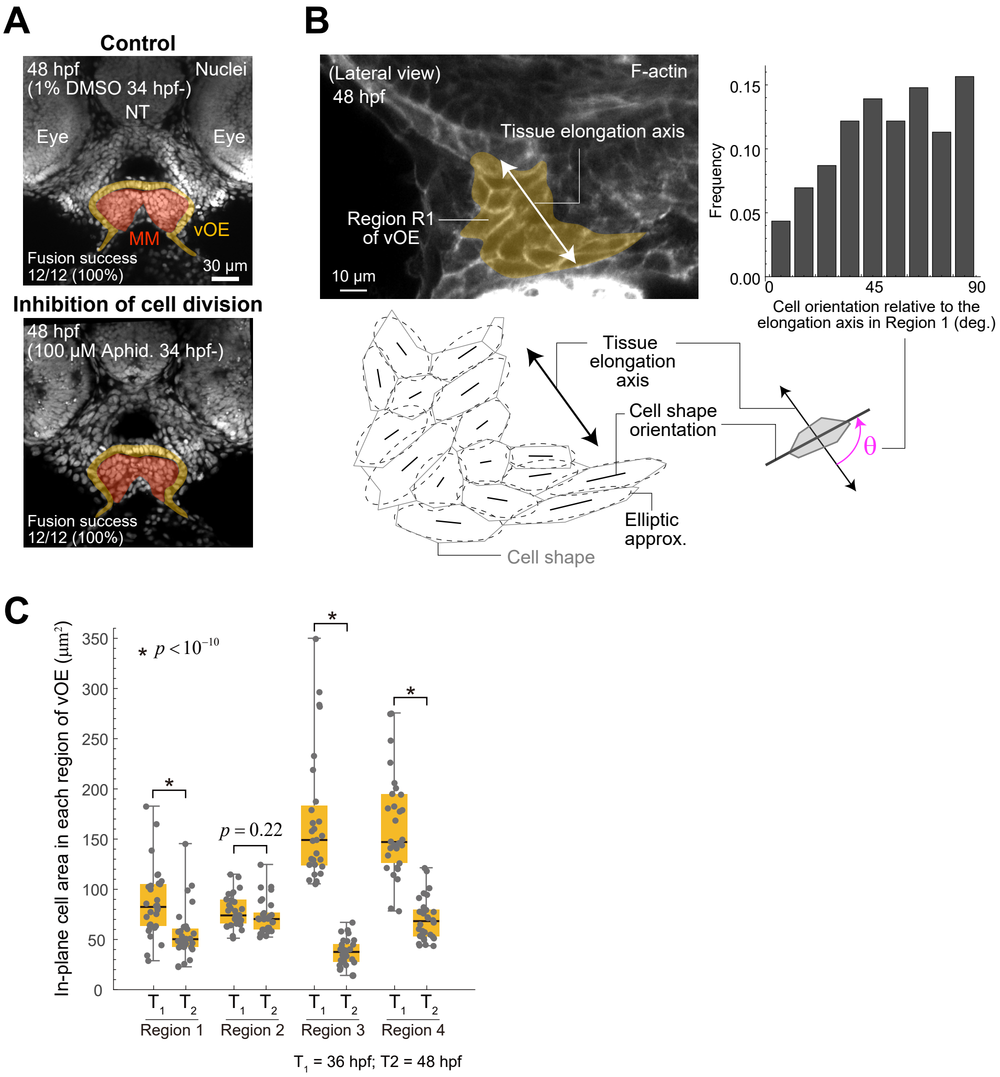

Fig. S4. Examination of the effects of cell division or cell shape change on ventral oral ectoderm tissue dynamics.

(**A**) Transverse section of embryos treated with DMSO (control, top) or aphidicolin (bottom). Folding of the ventral oral ectoderm (vOE, yellow) is observed in both conditions, indicating that cell division is not required for the lateral folding of vOE.

(**B**) Analysis of cell shape orientation in the anterolateral region (R1) of the vOE. (Top left) Cell shapes were visualized by phalloidin staining. The axis of tissue elongation was estimated from time-lapse imaging data of other embryos. R1, Region 1. Anterior is to the left. (Bottom) Quantification of cell shape orientation relative to the tissue elongation axis, based on segmentation and elliptical fitting of individual cells. (Top right) Histogram of cell shape orientation in R1 of the vOE (N = 5). The distribution is biased toward 90°, indicating a preferential orientation perpendicular to the tissue elongation axis. Taken together with the cell shape analysis at 36 hpf shown in Fig. 3D, these findings show that cell shape change is not a major contributor to the lateral folding of the vOE.

(**C**) Analysis of in-plane cell area in the four subregions that compose the vOE (see also Fig. 2D). Quantification of changes in cell area from 36 to 48 hpf across vOE subregions revealed a trend similar to that observed in the tissue-level deformation analysis, with region 3 exhibiting the most pronounced area shrinkage. For regions 1, 2, and 4, 30 cells from three embryos were analyzed at both 36 and 48 hpf; for region 3, 28 cells from three embryos at 36 hpf and 30 cells from three embryos at 48 hpf were analyzed. Statistical significance was assessed using Welch's t-test.

Figure S5
Hosoda et al.

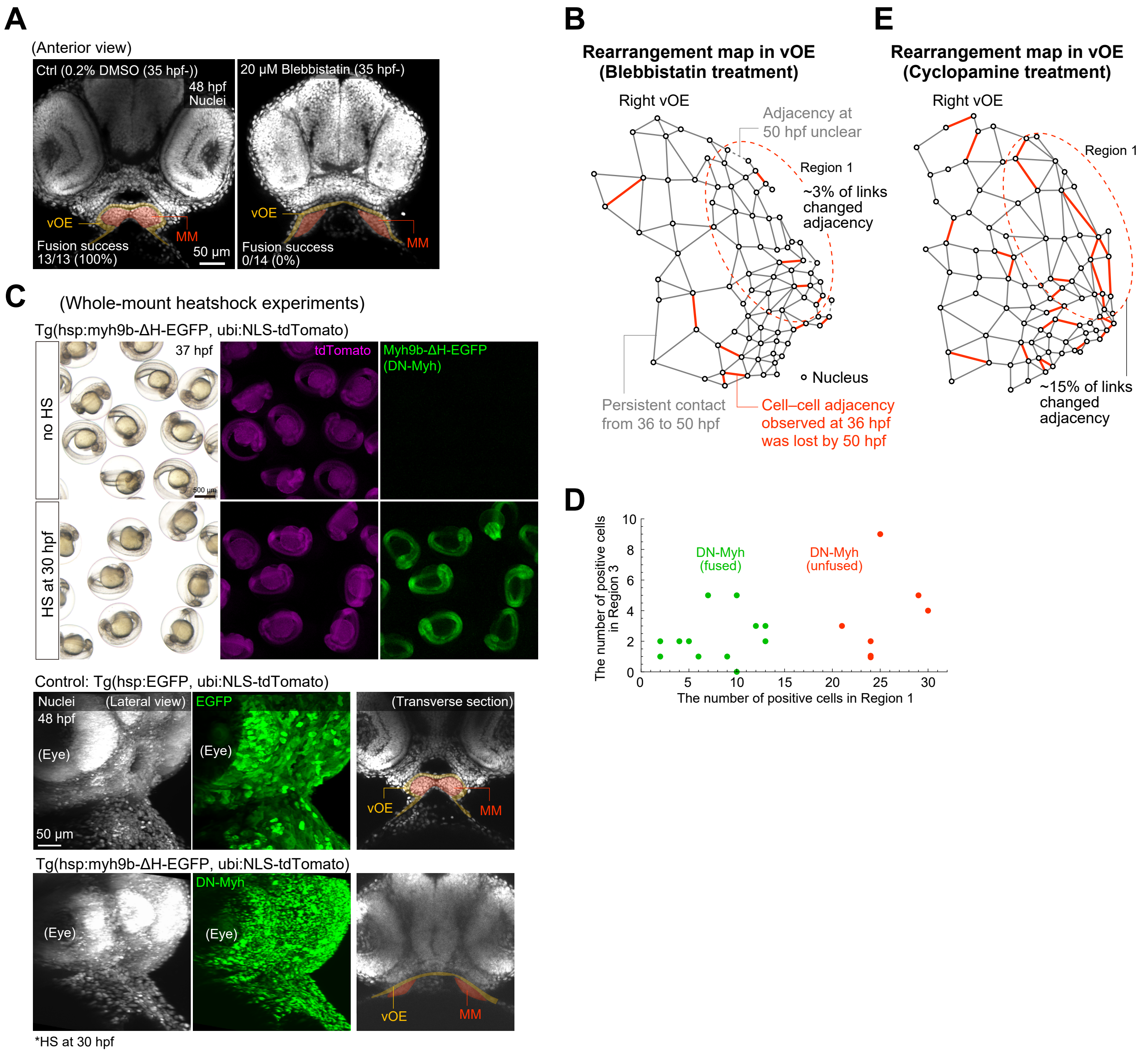

Fig. S5. Examination of phenotypes in embryos treated with a myosin inhibitor, Shh inhibitor, and in established transgenic (Tg) lines.

(**A**) Transverse section of embryos treated with DMSO (left, control) or (-)-blebbistatin (right). All embryos treated with (-)-blebbistatin, a non-muscle myosin II inhibitor, exhibited failure of lateral folding of the ventral oral ectoderm (vOE, yellow) and unfused mandibular mesenchyme (MM, red).
(**B**) Rearrangement map of the ventral oral ectoderm (vOE) in embryos treated with 20 μM (-)-blebbistatin from 35 hpf onward. Cell nuclei positions at 36 hpf are indicated by white circles. Only 3% of cell–cell contacts (represented as red links connecting cell centers) in Region 1 were rearranged by 50 hpf. Anterior is oriented toward the top.
(**C**) Whole-mount heatshock experiments examining the phenotypes of the Tg lines used in Fig. 4. (Top panels) Embryos of *Tg(hsp:myh9b-ΔH-EGFP, ubi:NLS-tdTomato)* heat-shocked at 30 hpf expressed a dominant-negative form of myosin heavy chain (DN-Myh) fused to EGFP, which was visualized by EGFP fluorescence. (Bottom panels) While the *Tg(hsp:EGFP, ubi:NLS-tdTomato)* control embryo heat-shocked at 30 hpf showed normal folding of the ventral oral ectoderm (vOE, yellow in transverse section) and fused mandibular mesenchyme (MM, red), the *Tg(hsp:myh9b-ΔH-EGFP, ubi:NLS-tdTomato)* embryo heat-shocked at 30 hpf exhibited deficiencies in both. Whereas non-fused EGFP was distributed diffusely throughout the entire cell, EGFP-tagged DN-Myh localized to the cell cortex and appeared as discrete puncta rather than being uniformly distributed.
(**D**) Quantification of DN-Myh-induced cells in region R1 (X-axis) and region R3 (Y-axis) of the vOE in the IR laser irradiation experiment shown in Fig. 4**C**. These results indicate that the number of DN-Myh–induced cells in R1 is critical for bilateral mandibular mesenchyme fusion, whereas leakage expression of DN-Myh in R3 does not affect fusion.
(**E**) Rearrangement map of the vOE in embryos treated with 100 μM cyclopamine from 24 hpf onward. Cell nuclei positions at 36 hpf are indicated by white circles. Approximately 15% of cell–cell contacts (represented as red links connecting cell centers) in Region 1 were rearranged by 50 hpf. Anterior is oriented toward the top.

Figure S6
Hosoda et al.

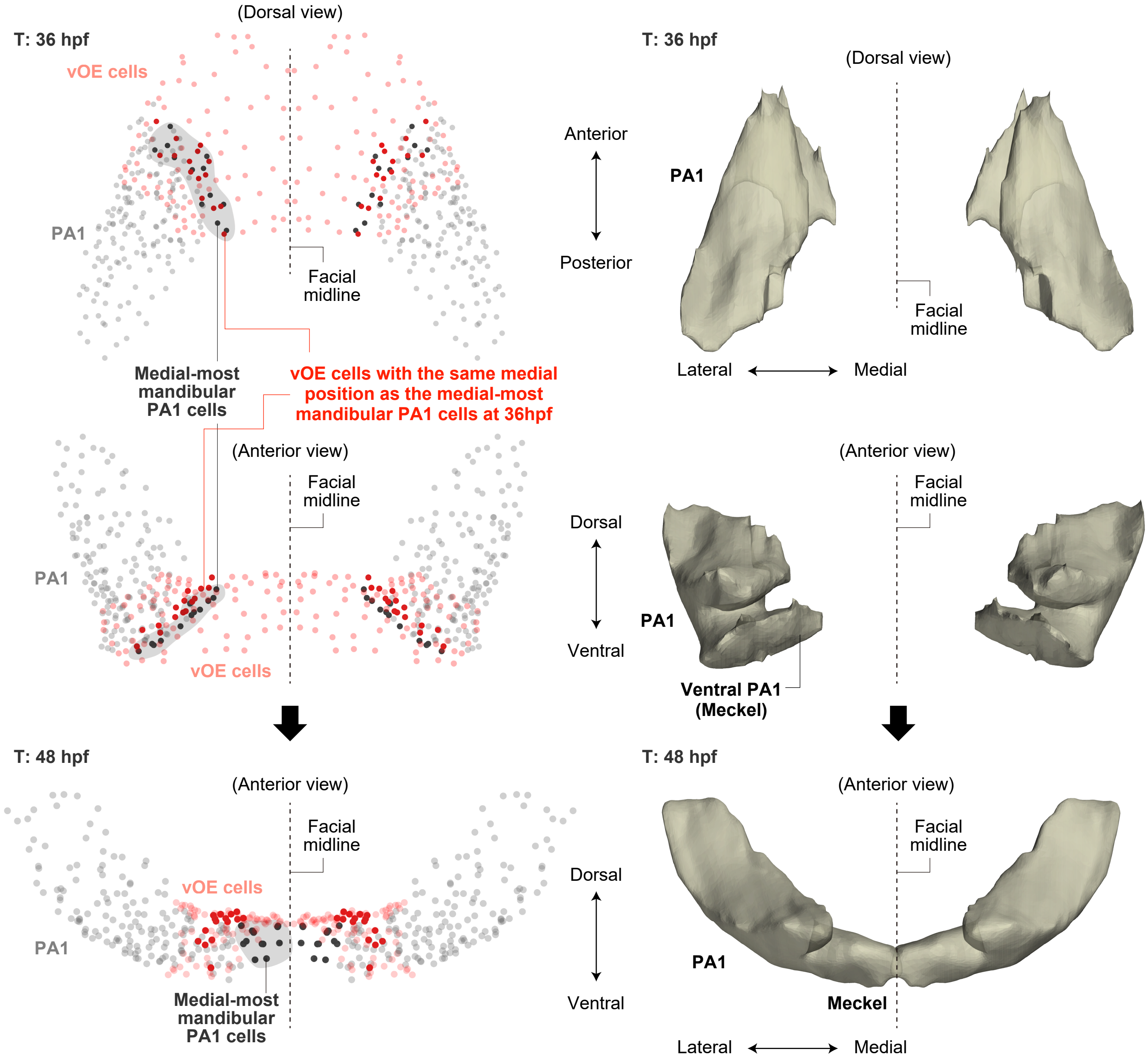

* For visualization, images from one side were mirrored and displayed together.

Fig. S6. Comparison of the movements of medial-most mandibular mesenchymal (MM) cells and adjacent ventral oral ectoderm (vOE) cells during elongation and fusion of the MM primordia.

PA1 mesenchymal cells (more precisely, neural crest–derived cells; gray dots) and vOE cells (red dots) were extracted from time-lapse imaging data. Medial-most MM cells at 36 hpf (prior to MM primordium elongation; dark gray dots) and vOE cells located adjacent to these cells at the same time point (dark red dots) were tracked, and their relative positions were examined at 48 hpf, corresponding to the stage of MM primordium fusion. As a result, MM cells were observed to move medially earlier than the overlying vOE cells. In addition, epithelial cells that were initially in contact with the medial-front mesenchymal cells before elongation were no longer adjacent to these cells at the time of primordium fusion, indicating that medial-most MM cells advance medially ahead of the overlying vOE cells.

Figure S7
Hosoda et al.

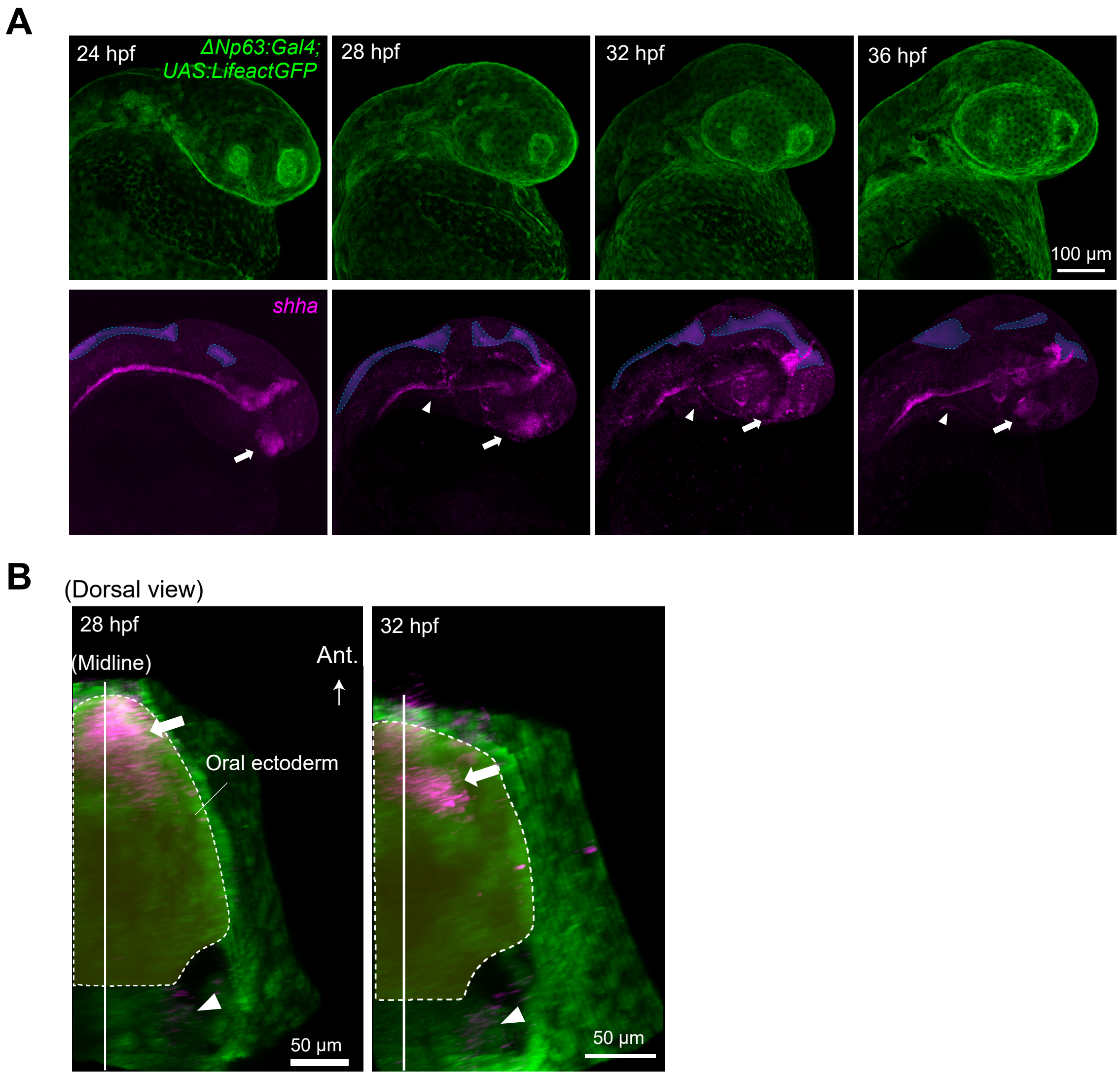

Fig. S7. *shha* expression patterns around the oral ectoderm.

(**A**) Whole-embryo views at 24, 28, 32, and 36 hpf. *shha* expression is stronger in the ventral neural tube than in surrounding tissues. Arrows indicate *shha* expression in the ventral diencephalon adjacent to the anterior oral ectoderm (OE). Weak *shha* expression was observed in endodermal epithelium near the second pharyngeal arch (PA2) (arrowheads). Brain ventricles outlined in cyan indicate nonspecific fluorescence.

(**B**) Dorsal-view subvolume images of the ventral forebrain and epithelia surrounding the OE (green) at 28 and 32 hpf. The domain of strong *shha* expression (magenta) in the ventral diencephalon (arrows) is closer to the anterior OE than *shha*-expressing domains in other surrounding tissues (e.g., the endoderm near PA2; arrowheads), which show weaker expression. Images are oriented with anterior (Ant.) at the top.

Probe oligo name	Sequence (5′-3′)
Drshha_set_01_N161_P1	GGTACGCGAaagcagcactctcgtcaaaagccgcat
Drshha_set_02_N161_P1	GGTACGCGAaagatgtcttcttctgccgtagcctct
Drshha_set_03_N161_P1	GGTACGCGAaatccgcgacattaggtatgaactgct
Drshha_set_04_N161_P1	GGTACGCGAaactcttccctcgtagtggagtgattc
Drshha_set_05_N161_P1	GGTACGCGAaagacagtgtcccgtatttgctcttgt
Drshha_set_06_N161_P1	GGTACGCGAaagaacaccaggtttcccgcgctgtct
Drshha_set_07_N161_P1	GGTACGCGAaaaaaacacacgtcgcgtcgtggagtc
Drshha_set_08_N161_P1	GGTACGCGAaatgagcggcggtgagggtgatctttt
Drshha_set_09_N161_P1	GGTACGCGAaagctctgacactgctggcatacgcgg
Drshha_set_10_N161_P1	GGTACGCGAaagatgacagatttaagctggccgcta
Drshha_set_11_N161_P1	GGTACGCGAaatgtgcagtcactggtgcgaacgagc
Drshha_set_12_N161_P1	GGTACGCGAaacgggcgcgaaggccaaatgcgcaag
Drshha_set_13_N161_P1	GGTACGCGAaactgctggagttttgggggaacagga
Drshha_set_14_N161_P1	GGTACGCGAaaaggagcctggagtaccagtggaccc
Drshha_set_01_N161_P2	ccaaggacagagtgagaagagacacaaAGGTAGGTGTAA
Drshha_set_02_N161_P2	tgtaggcgagaggtgtcagcttcttaaAGGTAGGTGTAA
Drshha_set_03_N161_P2	tatctgccgctggcccctaaggtctaaAGGTAGGTGTAA
Drshha_set_04_N161_P2	ctcggtcagaggtggtaatatcaacaaAGGTAGGTGTAA
Drshha_set_05_N161_P2	tcaaatccagcctccacagctaggcaaAGGTAGGTGTAA
Drshha_set_06_N161_P2	tcggtctgtgaacatgatgaagtcgaaAGGTAGGTGTAA
Drshha_set_07_N161_P2	caacgggttcttgcgtttctatgacaaAGGTAGGTGTAA
Drshha_set_08_N161_P2	gttgagttgtcgaggacaaaaaggaaaAGGTAGGTGTAA
Drshha_set_09_N161_P2	tcatcaacaaccatcaccttttgtcaaAGGTAGGTGTAA
Drshha_set_10_N161_P2	ctgctcctccgtgtatatccgctgcaaAGGTAGGTGTAA
Drshha_set_11_N161_P2	agtattctgtcgaccacaatggtccaaAGGTAGGTGTAA
Drshha_set_12_N161_P2	atgatgacacgtaataatacagcctaaAGGTAGGTGTAA
Drshha_set_13_N161_P2	tcctgttgtaaagtcgcattggaccaaAGGTAGGTGTAA
Drshha_set_14_N161_P2	tccaaaagccacgttcccatttgataaAGGTAGGTGTAA
Drptch2_set_01_N161_P1	GGTACGCGAaaggggggtaaatctccaaaaacaccac
Drptch2_set_02_N161_P1	GGTACGCGAaaaaaagagaaaagcctggaacctcgc
Drptch2_set_03_N161_P1	GGTACGCGAaagtagctccttgctcactcgactgcc
Drptch2_set_04_N161_P1	GGTACGCGAaacccggtaagtaggcggagcctccct
Drptch2_set_05_N161_P1	GGTACGCGAaatctgtgtcactgggatctaagcatg
Drptch2_set_06_N161_P1	GGTACGCGAaaatgtcatggatctcatagtcatctt
Drptch2_set_07_N161_P1	GGTACGCGAaaattaaaggacagtcccaacagcgaa
Drptch2_set_08_N161_P1	GGTACGCGAaagccatgaagaaagcaatcatgttat
Drptch2_set_09_N161_P1	GGTACGCGAaatgagagctcttgaggctggatttga
Drptch2_set_10_N161_P1	GGTACGCGAaaatatctgggaggtgggtggcaagat
Drptch2_set_11_N161_P1	GGTACGCGAaaggggcatacttttctcttgcaaagc
Drptch2_set_12_N161_P1	GGTACGCGAaagccatccatggtaaccaggtacata
Drptch2_set_13_N161_P1	GGTACGCGAaacgatagctgtcataggtaattttgc
Drptch2_set_14_N161_P1	GGTACGCGAaaggatcattactgacccatactgtca
Drptch2_set_15_N161_P1	GGTACGCGAaatgaggcttgtcttagtccatttagg
Drptch2_set_16_N161_P1	GGTACGCGAaagataccaattaggcccataatgcca
Drptch2_set_17_N161_P1	GGTACGCGAaagtcaatcacaggagcaaacatatgt
Drptch2_set_18_N161_P1	GGTACGCGAaacaggagaaggactttgcaaatgatt

Drptch2_set_19_N161_P1	GGTACGCGAaatgtcacagtacttgtattcctcctc
Drptch2_set_20_N161_P1	GGTACGCGAaatctgtgagctgatggagttgacagc
Drptch2_set_01_N161_P2	acaggcggcggagagcgtgtgtaacaaAGGTAGGTGTAA
Drptch2_set_02_N161_P2	agtgtcgctggatgtgacagcccagaaAGGTAGGTGTAA
Drptch2_set_03_N161_P2	cctctccttgcttctcttttgtgtaaaAGGTAGGTGTAA
Drptch2_set_04_N161_P2	agattcatccattgaatgtcaggcaaaAGGTAGGTGTAA
Drptch2_set_05_N161_P2	tccttattaggggcactgtgagggcaaAGGTAGGTGTAA
Drptch2_set_06_N161_P2	gcggtggctttgtcctcattccagtaaAGGTAGGTGTAA
Drptch2_set_07_N161_P2	aaatggtagcacctgcgtggtggcgaaAGGTAGGTGTAA
Drptch2_set_08_N161_P2	cgaagagcaggaattggcactagggaaAGGTAGGTGTAA
Drptch2_set_09_N161_P2	agcacgctgatggttgtcattagcaaaAGGTAGGTGTAA
Drptch2_set_10_N161_P2	ataggaccatggatggaggagtggtaaAGGTAGGTGTAA
Drptch2_set_11_N161_P2	gtcttagtttcaggcttcagcaacaaaAGGTAGGTGTAA
Drptch2_set_12_N161_P2	ctgtctttgagaacgggcatagtcaaaAGGTAGGTGTAA
Drptch2_set_13_N161_P2	gccagtgctccatcctctgtgccataaAGGTAGGTGTAA
Drptch2_set_14_N161_P2	ttggcttgggaggcagcatagcccaaaAGGTAGGTGTAA
Drptch2_set_15_N161_P2	cacgctctcgatggcttcaataaagaaAGGTAGGTGTAA
Drptch2_set_16_N161_P2	gatgaccacaggaatagcactcagcaaAGGTAGGTGTAA
Drptch2_set_17_N161_P2	cactcccaaaagagtggagatggcgaaAGGTAGGTGTAA
Drptch2_set_18_N161_P2	ggtggttcataggaggaggcatgggaaAGGTAGGTGTAA
Drptch2_set_19_N161_P2	gagctggaggtgtgatgtatgtgctaaAGGTAGGTGTAA
Drptch2_set_20_N161_P2	cctcattttattgtcccaccgtttaaAGGTAGGTGTAA

Table S1. Probe oligos for in situ HCR.

Movie S1. Morphogenesis of the first pharyngeal arch (PA1) with cell trajectories.

Time-lapse imaging showing PA1 morphogenesis in a *Tg(sox10:EGFP)* embryo from 36 to 48 hpf. Nuclei were labeled by tdTomato-NLS mRNA injection at the one-cell stage. The imaging interval is 30 min. (Top) Anterolateral view. Nuclei are shown in white. Tracked PA1 cells (N = 163 cells at 36 hpf) are indicated as magenta particles positioned near the centers of nuclei, representing cell positions. For clarity, only the tracked particles are shown alone in the right panel. (Bottom) Ventral view, shown after the anterolateral view (top). Mirrored copies of particles representing cell positions at all time points were generated across the midplane. Particles corresponding to each subdivision of PA1—presumptive Meckel's cartilage (Meckel, red), presumptive palatoquadrate cartilage (PQ, cyan), dorsoanterior (maxillary prominence, green), and dorsoposterior (yellow) domains—are labeled in the movie.

Movie S2. Anterolateral folding dynamics of the ventral oral ectoderm (vOE) wrapping the mandibular mesenchymal primordium.

Time-lapse imaging showing the anterolateral folding dynamics of the vOE, leading to wrapping of the mandibular mesenchymal primordium from 36 to 48 hpf. Nuclei (white) were labeled by tdTomato-NLS mRNA injection at the one-cell stage. Tracked mandibular PA1 (light yellow) and vOE (dark orange) cells are indicated as particles positioned near the centers of nuclei, representing cell positions. Polygons representing PA1 (light yellow), AA1 (brown), and a light-gray open surface polygon representing the basal epidermis continuous with the ventral oral ectoderm are shown at 36 and 48 hpf. A bright orange open surface polygon reconstructed from vOE cell particle positions represents the ventral oral ectoderm structure. The imaging interval is 30 min. The view is rotated during the movie to facilitate visualization of the three-dimensional tissue and organ arrangement and the anterolateral folding dynamics of the vOE.
Abbreviations: AA1, aortic arch 1; PA1, first pharyngeal arch; vOE, ventral oral ectoderm.

Movie S3. Anterolateral folding dynamics of the ventral oral ectoderm (vOE), highlighting cells in the anterolateral region (R1).

Time-lapse imaging showing the anterolateral folding behavior of the vOE from 36 to 48 hpf. Tracked vOE cells are indicated as dark orange particles positioned near the centers of nuclei, representing nuclear-based cell positions. An open surface polygon (light orange) reconstructed from cell particle positions represents the ventral oral ectoderm structure. Cells in the anterolateral region (R1) are indicated in red. Two small populations of cells arranged along the anterior–posterior (A–P) axis (green) and along the medial–lateral (M–L) axis (cyan) at 36 hpf are also indicated. The midplane defined at 48 hpf is shown. The imaging interval is 30 min.
